# *LIG4* mediates Wnt signalling-induced radioresistance

**DOI:** 10.1038/ncomms10994

**Published:** 2016-03-24

**Authors:** Sohee Jun, Youn-Sang Jung, Han Na Suh, Wenqi Wang, Moon Jong Kim, Young Sun Oh, Esther M. Lien, Xi Shen, Yoshihisa Matsumoto, Pierre D. McCrea, Lei Li, Junjie Chen, Jae-Il Park

**Affiliations:** 1 Department of Experimental Radiation Oncology, The University of Texas MD Anderson Cancer Center, Houston, Texas 77030, USA; 2 Research Laboratory for Nuclear Reactors, Tokyo Institute of Technology, Tokyo 152-8550, Japan; 3 Department of Molecular Genetics, The University of Texas MD Anderson Cancer Center, Houston, Texas 77030, USA; 4 Graduate School of Biomedical Sciences at Houston, The University of Texas Health Science Center and MD Anderson Cancer Center, Houston, Texas 77030, USA; 5 Program in Genes and Development, The University of Texas MD Anderson Cancer Center, Houston, Texas 77030, USA

## Abstract

Despite the implication of Wnt signalling in radioresistance, the underlying mechanisms are unknown. Here we find that high Wnt signalling is associated with radioresistance in colorectal cancer (CRC) cells and intestinal stem cells (ISCs). We find that *LIG4*, a DNA ligase in DNA double-strand break repair, is a direct target of β-catenin. Wnt signalling enhances non-homologous end-joining repair in CRC, which is mediated by *LIG4* transactivated by β-catenin. During radiation-induced intestinal regeneration, LIG4 mainly expressed in the crypts is conditionally upregulated in ISCs, accompanied by Wnt/β-catenin signalling activation. Importantly, among the DNA repair genes, LIG4 is highly upregulated in human CRC cells, in correlation with β-catenin hyperactivation. Furthermore, blocking LIG4 sensitizes CRC cells to radiation. Our results reveal the molecular mechanism of Wnt signalling-induced radioresistance in CRC and ISCs, and further unveils the unexpected convergence between Wnt signalling and DNA repair pathways in tumorigenesis and tissue regeneration.

## Abstract (web summary)

The Wnt/β-catenin signalling pathway contributes to radio resistance in intestinal stem cells but the underlying mechanism is currently unknown. In this study, the authors demonstrate that LIG4, a DNA ligase involved in the DNA repair process, is a direct target of β-catenin and it specifically mediates non-homologous end joining repair in colorectal cancer cells.

## Graphical abstract

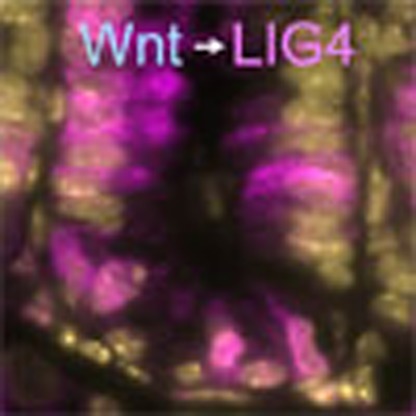

Wnt signalling is essential for stem cell regulation in development and tissue homeostasis^1^. Wnt ligands bind to Frizzled receptors and low-density lipoprotein receptor-related protein 5/6 co-receptors, which stabilizes β-catenin protein by inhibiting the protein destruction complex composed of adenomatous polyposis coli, Axin, casein kinase 1 and glycogen synthase kinase 3. Subsequently, the stabilized β-catenin protein is translocated into the nucleus and replaces T-cell factor (TCF)-associated co-repressors with coactivators, which results in the transcriptional activation of the β-catenin target genes^2^.

Deregulation of Wnt/β-catenin signalling leads to human cancers^3^. For example, frequent genetic mutations in Wnt signalling components have been closely associated with human colorectal cancer (CRC)^4^. In mouse models, genetic mutation leading to the hyperactivation of Wnt signalling induced mammary tumours and intestinal adenomas^5^.

Gastrointestinal tissue is often damaged by ionizing radiation (IR) during cancer therapeutic procedures or by nuclear accidents. Patients undergoing radiotherapy can experience radiation-induced gastrointestinal syndrome (RIGS), which involves death of intestinal epithelial cells (IECs) and subsequent villous blunting and fusion^6^. Chronic RIGS results in intestinal inflammation, mucosal thickening and fibrosis; acute RIGS leads to malabsorption, electrolyte imbalance, diarrhoea, weight loss and potential death (within 10 days of IR exposure)^7^.

In the intestinal epithelium, terminally differentiated IECs are constitutively replaced by newly divided IECs from intestinal stem cells (ISCs) located in the crypts. This biological process is tightly controlled by Wnt signalling^8^. On IR treatment, cells in the crypts undergo acute apoptosis or stop cell division. Depending on the IR dosage, surviving clonal stem/progenitor cells regenerate the crypts and subsequently rebuild the villi. Recently, several reports suggested that Wnt signalling prevents IR damage in various tissues, including the salivary gland cells^9^,^10^, mammary gland cells^11^, IECs^12^,^13^, bone marrow cells^14^ and osteoblasts^15^. Other studies have also shown that active Wnt signalling induces radioresistance in several human cancers, including head and neck cancer^16^, breast cancer^17^,^18^,^19^, nasopharyngeal cancer^20^, oesophageal cancer^21^,^22^, glioblastoma^23^ and CRC^24^. However, it remains undetermined how Wnt signalling contributes to radioresistance in normal and cancer cells.

DNA double-strand breaks (DSBs) induce harmful lesions, which causes cell-cycle arrest or cell death. DSBs are generated by exogenous factors including IR or endogenously produced during genetic recombination of immune receptor genes^25^. DSBs are repaired by two major pathways: homologous recombination (HR) and non-homologous end joining (NHEJ)^26^. NHEJ is the predominant process for DSB repair. While HR is active during S and G2 phases of the cell cycle^27^, NHEJ occurs throughout the cell cycle. In the process of NHEJ, the Ku70/80 heterodimer recognizes DSBs, as the DNA-PK complex composed of Ku70/Ku80 and DNA-PK_CS_ (DNA-dependent protein kinase catalytic subunit). In association with XRCC4 (X-ray cross-complement protein 4) and XLF (XRCC4-like factor; also called NHEJ1/Cernunnos), DNA ligase IV (LIG4) completes DSB end joining^28^. Recently, PAXX (paralogue of XRCC4 and XLF) was identified as a new component of NHEJ process, in playing a role in promoting Ku-dependent DNA ligation and the assembly of core–NHEJ components^29^.

*LIG4* is a gene that encodes ATP-dependent DNA ligase IV in the repair of DSBs. LIG4 syndrome is caused by mutations in *LIG4* and involves reduced DNA ligase activity^30^. In humans, LIG4 syndrome is characterized by radiosensitivity, microcephaly, neurological abnormalities, bone marrow failure and increased cancer susceptibility^30^. Similarly, *LIG4* somatic knockout cell lines display extreme radiosensitization^31^, suggesting that *LIG4* is indispensable to DSB repair. Several studies showed that mutation in *LIG4* impaired the biological functions of tissue stem cells and induced pluripotent cells^32^,^33^,^34^, indicating the essential roles of DNA repair in the maintenance of stemness^25^.

Accumulating evidence suggests that tissue stem cells and tumour-initiating cells (TICs) are relatively resistant to genotoxic stress, which possibly contributes to the maintenance of genomic stability; however, the mechanism of this resistance is unknown. For example, mutation frequency is lower in embryonic stem cells (ESCs) than in differentiated cells^35^,^36^, with increased DNA repair activity^37^,^38^. Similarly, many studies have suggested that TICs are resistant to genotoxic stress in various human cancers^18^,^39^,^40^,^41^. Despite the implication of Wnt signalling in radioresistance in stem cells and TICs, the molecular mechanism remains undetermined. Here we investigated how Wnt signalling is associated with radioresistance. Our results show that Wnt/β-catenin signalling enhances *LIG4* expression, and upregulated LIG4 plays a key role in radioresistance in tissue stem cells and cancer cells.

## Results

### Wnt signalling-induced radioresistance

**Figure 1:**
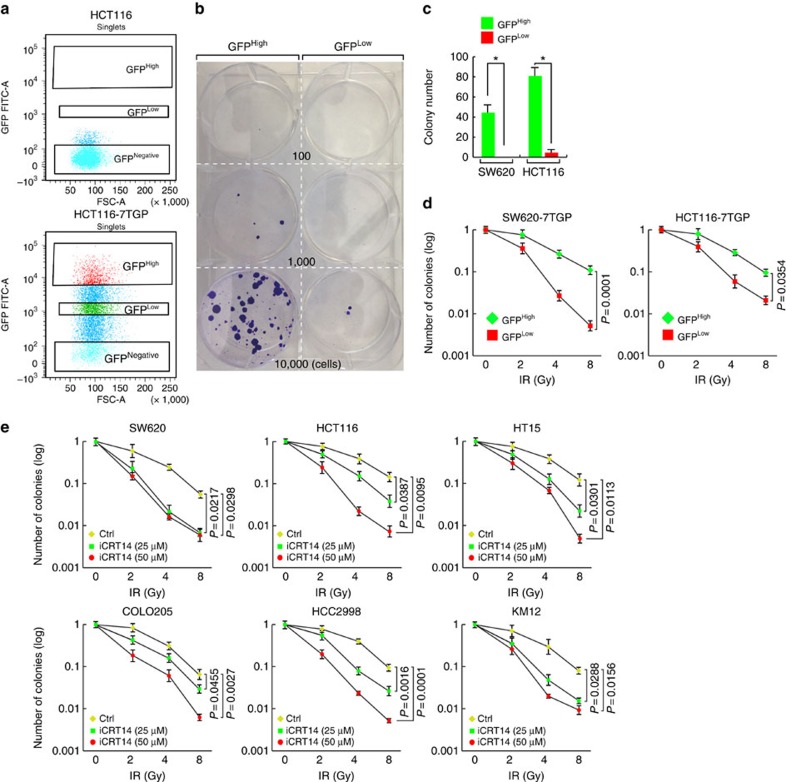
Association of Wnt signalling and radioresistance in CRC cells. (**a**) CRC cell sorting by Wnt signalling activity. HCT116-7TGP cells were sorted based on GFP expression (GFP^High^ and GFP^Low^) by FACS. HCT116 parental cells served as a negative control for cell sorting. (**b**,**c**) Increased cell survival in GFP^High^ than GFP^Low^ cells. HCT116-7TGP (GFP^High^ and GFP^Low^) cells were treated with IR (4 Gy). Two weeks later, cells were fixed for crystal violet staining (**b**). Colony number quantification of HCT116-7TGP and SW620-7TGP cells after IR (4 Gy) (**c**). *P<0.05. Student's *t*-test; *N*=3; error bars=±s.e.m. (**d**) Radioresistance in GFP^High^ CRC cells compared with GFP^Low^ cells. Clonogenic cell survival assay of SW620-7TGP and HCT116-7TGP cells. After IR (2, 4 and 8 Gy), sorted cells (GFP^High^ and GFP^Low^) were seeded based on plating efficiency. Two weeks later, the number of colonies was quantified. Student's *t*-test; *N*=3; error bars=±s.e.m. (**e**) Radiosensitization of CRC cells by iCRT14. CRC cells (SW620, HCT116, HT15, COLO205, HCC2998 and KM12) were pretreated with iCRT14 (24 h) and IR (0, 2, 4 and 8 Gy). Two weeks later, colonies were counted for quantification. Student's *t*-test; *N*=3; error bars=±s.e.m.

While Wnt signalling contributes to radioresistance in cancer, Wnt signalling activity varies among different types of cancer, which prompted us to hypothesize that increased Wnt signalling activity causes increased radioresistance. To test this hypothesis, we first isolated CRC cells based on Wnt signalling activity. We subjected CRC cells to stable transduction with 7TGP, a lentivirus encoding β-catenin green fluorescent protein (GFP) reporter^42^. After viral transduction, we sorted CRC cells using fluorescence-activated cell sorting (FACS) on the basis of GFP expression (Fig. 1a and Supplementary Fig. 1A). By immunofluorescent (IF) staining, we confirmed the differential expression of GFP (GFP^High^ and GFP^Low^; Supplementary Fig. 1B,C). Compared with GFP^Low^ cells, GFP^High^ cells expressed relatively high levels of β-catenin and its target genes, *CD44* and *CD133*, mirroring high Wnt signalling activity (Supplementary Fig. 1D–F). Owing to the high Wnt signalling activity in colon cancer stem cells^43^,^44^,^45^,^46^,^47^, we also investigated whether high Wnt signalling activity is associated with increased sphere formation, an indirect indicator of stemness. Indeed, under non-adherent cell culture conditions, GFP^High^ cells had more and larger colonies than did GFP^Low^ cells (Supplementary Fig. 1G,H), indicating that GFP^High^ cells possess increased cell stemness. Next, using the GFP^High^ and GFP^Low^ CRC cells, we investigated whether differential Wnt signalling activity is associated with different radiosensitivity. Interestingly, cell survival assays showed that GFP^High^ HCT116 and GFP^High^ SW620 cells were more resistant to IR (4 Gy) than GFP^Low^ cells (Fig. 1b,c). It is noteworthy that GFP^High^ cells did not show the differences in cell proliferation, compared with GFP^Low^ cells (Supplementary Fig. 2), which excludes the possibility that cell proliferation might affect cell survival on radiation treatment. Next, we performed clonogenic cell survival assays. GFP^High^ and GFP^Low^ HCT116 and SW620 cells were treated with IR (2, 4 and 8 Gy). Colonies were quantified after 14 days. We found that GFP^High^ CRC cells are more resistant to IR than GFP^Low^ cells (Fig. 1d). Conversely, we also tested whether inhibiting Wnt signalling sensitizes CRC cells to IR. We pretreated CRC cells with iCRT14, a specific inhibitor of β-catenin–TCF interaction, and subsequently exposed cells to IR. Intriguingly, iCRT14 treatment (25 and 50 μM), sensitized CRC cells (SW620, HCT116, HT15, COLO205, HCC2998 and KM12) to IR (Fig. 1e). Of note is that iCRT14 *per se* (up to 75μM) did not impair CRC cell proliferation (Supplementary Fig. 3), excluding the possibility that iCRT14-induced cell growth inhibition affects radiosensitization results. Moreover, we questioned whether iCRT14 sensitizes GFP^High^ CRC cells to IR. Indeed, iCRT14-treated GFP^High^ HCT116 and GFP^High^ SW620 cells became radiation-sensitive (Supplementary Fig. 4). These results suggest that β-catenin contributes to radioresistance in CRC cells.

### *LIG4* transactivation by Wnt/β-catenin signalling

**Figure 2:**
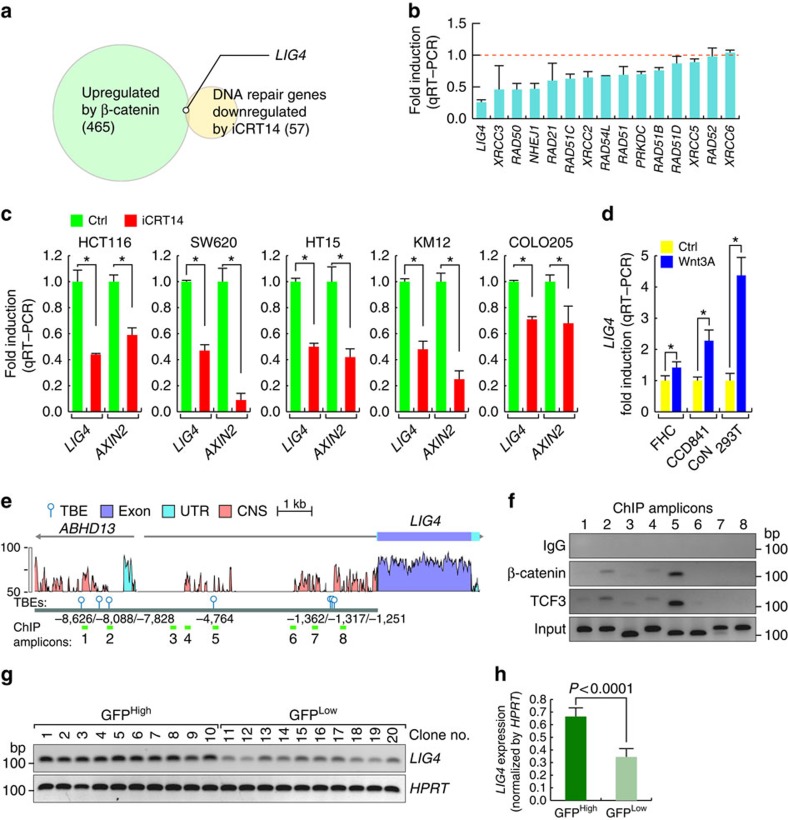
*LIG4* transactivation by β-catenin. (**a**) Identification of β-catenin target genes. DNA repair genes that are upregulated by β-catenin (465 genes) and downregulated by iCRT14 (50 μM for 24 h; 57 genes) were identified. Notably, *LIG4* is both upregulated by β-catenin and downregulated by iCRT14. Fold change > 2; *P*<0.005. Student's *t*-test; *N*=3. (**b**) Identification of NHEJ genes downregulated by β-catenin inhibition. HCT116 cells were treated with iCRT14 (50 μM for 24 h) and analyzed for expression of NHEJ repair genes using qRT-PCR. Student's *t*-test; *N*=3; error bars=±SEM. (**c**) Downregulation of *LIG4* by iCRT14 in CRC cells. CRC cells (HCT116, SW620, HT15, KM12 and COLO205) were treated with iCRT14 (50 μM for 24h) and analyzed by qRT-PCR. *AXIN2* served as a positive control for the β-catenin target gene. Student's *t*-test; *N*=3; *P<0.05; error bars=±s.e.m. (**d**) Upregulation of *LIG4* by Wnt3A in IECs. Human IECs (FHC and CCD841CoN) and 293T cells were treated with Wnt3A (200 ng ml^−1^ for 24 h) and analysed by qRT–PCR. Student's *t*-test; *N*=3; *P<0.05; error bars=±s.e.m. (**e**) *LIG4* promoter analysis. Conserved noncoding sequences (CNS) found in both the mouse and human *LIG4* promoter were analysed for potential TCF/LEF-binding elements (TBEs; balloons). UTR: untranslated region. (**f**) β-catenin transcriptional complex occupies *LIG4* promoter. HCT116 cells were analysed by ChIP assays. ChIP amplicons (1–8) were detected by ChIP-PCR. (**g**,**h**) Upregulation of LIG4 in GFP^High^ cells. SW620-7TGP CRC cells were sorted into GFP^High^ and GFP^Low^ cells, and clonally selected for semi-quantitative RT–PCR of *LIG4* expression (**g**). ImageJ analysis of *LIG4* expression normalized by hypoxanthine phosphoribosyltransferase 1 (*HPRT*) (**h**). Student's *t*-test; *N*=3; *P<0.05; error bars=±s.e.m.

Next, we sought to understand the molecular mechanism of Wnt signalling-induced radioresistance. Wnt signalling mainly controls cellular physiology by β-catenin-mediated transactivation of Wnt target genes. Thus, we hypothesized that β-catenin modulates DNA repair genes. To test this hypothesis, we performed cDNA microarrays and PCR arrays. We identified genes that were upregulated with a fold change of two or greater by β-catenin ectopic expression (cDNA microarray; gain of function) and also downregulated by iCRT14 (PCR array; loss of function). Of the genes involved in DNA repair pathways, we found that *LIG4* expression was upregulated by β-catenin (Fig. 2a and Supplementary Data 1,2,3). Subsequent quantitative reverse transcription–PCR (qRT–PCR) analysis results confirmed that iCRT14 treatment significantly downregulated the transcriptional level of *LIG4* in HCT116 cells (Fig. 2b). We further confirmed that both *LIG4* and *AXIN2*, well-established β-catenin target genes, were downregulated by iCRT14 in other CRC cells (SW620, HT15, KM12 and COLO205; Fig. 2c). Conversely, Wnt3A treatment upregulated *LIG4* expression in IECs (FHC and CCD841CoN) and 293T cells (Fig. 2d). These results suggest that β-catenin induces *LIG4* upregulation.

Next, we investigated whether β-catenin transactivates *LIG4* expression. We identified seven consensus TCF-binding elements (TBE; CTTTGA/TA/T) within 10-kb upstream of the transcription start site of the *LIG4* gene. *In silico* analysis using VISTA genome browser located TBEs in conserved noncoding sequences (CNS) of both the human and mouse *LIG4* promoter (Fig. 2e), implying that TBEs governing *LIG4* transactivation might be evolutionarily conserved in mammals. Next, we performed chromatin immunoprecipitation (ChIP) promoter scanning. We designed eight pairs of ChIP-PCR primers and examined whether β-catenin and TCF3 bind to TBEs. We found that β-catenin and TCF3 co-occupy TBEs at −8,088 and −4,764 of the *LIG4* promoter in HCT116 cells (Fig. 2f).

To determine whether Wnt signalling activity is associated with the expression of *LIG4*, we next analysed the expression of LIG4 in clonally selected CRC cells (GFP^High^ and GFP^Low^ SW620-7TGP). Semi-quantitative RT–PCR showed that GFP^High^ cells express relatively higher level of *LIG4*, compared with that in GFP^Low^ cells (Fig. 2g,h). These results suggest that β-catenin upregulates *LIG4* expression in CRC cells and IECs.

### LIG4 mediates Wnt signalling-induced radioresistance

**Figure 3:**
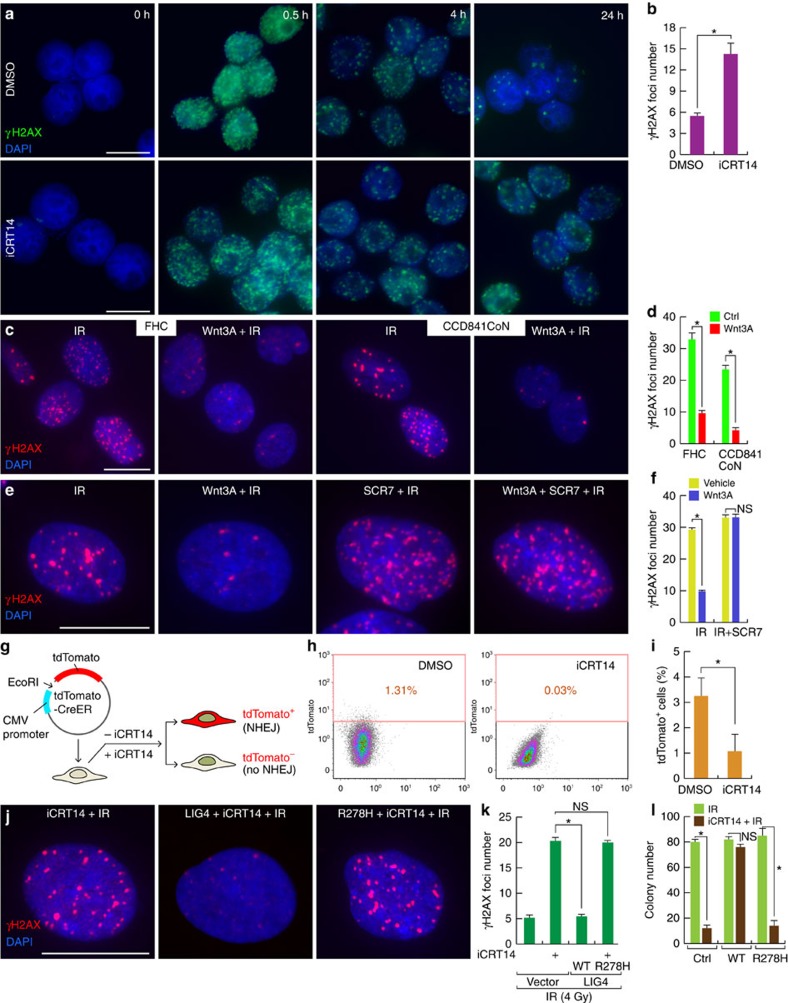
LIG4 mediates Wnt signalling-induced radioresistance. (**a**,**b**) Increase of unrepaired DNA damage by Wnt signalling inhibition. HCT116 cells were pretreated with dimethylsulfoxide (DMSO; vehicle control) or iCRT14 (50μM for 24h) and were treated with IR (4 Gy). At each time point, cells were collected for IF staining for phospho-γH2AX (A); quantitative analysis of DNA damage foci (**b**). Student's *t*-test; *N*=3; *P<0.05; error bars=±s.e.m.; scale bars=20μm. (**c**,**d**) Decreased DNA damage foci formation by Wnt signalling. Human IECs (FHC and CCD841CoN) were pretreated with Wnt3A (200 ng ml^−1^ for 24 h) and were subjected to IR (4 Gy). After 24 h, cells were analysed for DNA damage foci formation. IF staining (**c**); quantitative analysis (**d**). Student's *t*-test; *N*=3; *P<0.05; error bars=±s.e.m.; scale bars=20μm. (**e**,**f**) Inhibition of LIG4 blocks Wnt3A-induced radioresistance. CCD841CoN IECs were pretreated with Wnt3A (200 ng ml^−1^ for 24 h) and subjected to IR (4 Gy) in the absence or presence of SCR7 (10 μM); quantitative analysis (**f**). Student's *t*-test; *N*=3; *P<0.05; error bars=±s.e.m.; scale bars=20μm. (**g**–**i**) Reduced NHEJ activity after Wnt signalling inhibition. SW620-7TGP GFP^High^ cells were co-transfected with linearized tdTomato-expressing plasmids (**g**) CMV, cytomegalovirus. Twenty-four hours after transfection, cells were analysed by FACS (**h**) and were quantified (**i**). Student's *t*-test; *N*=3; *P<0.05; NS (not significant, *P*≥0.05); error bars=±s.e.m. (**j**–**l**) LIG4 expression rescues Wnt signalling inhibition-induced radiosensitization. iCRT14-treated HCT116 cells (control: empty vector; stably expressing wild-type (WT) LIG4 or R278H mutant LIG4) were exposed to IR (4 Gy). After 24 h, cells were analysed for γH2AX foci formation; IF staining (**j**), statistical analysis (**k**) and crystal violet staining (14 days) (**l**). Student's *t*-test; *N*=3; *P<0.05; NS (not significant, *P*≥0.05); error bars=±s.e.m.; scale bars=20μm. For **b**,**d**,**f** and **k**, the number of phospho-γH2AX foci were counted in more than 20 nuclei from the three biological replicas.

With XRCC4 and XLF, LIG4 participates in DSB repair through NHEJ. *LIG4* somatically targeted (*LIG4*^−/−^) HCT116 cells are viable but are highly sensitive to DNA genotoxic stress^31^, suggesting that LIG4 plays a crucial role in DSB repair in CRC cells. Owing to the transactivation of *LIG4* by β-catenin, we asked whether LIG4 mediates Wnt/β-catenin signalling-induced radioresistance in CRC cells. We assessed phosphorylated γH2AX, a surrogate marker of DNA DSBs, to determine whether γH2AX foci formation is inversely associated with Wnt signalling activity. We treated HCT116 cells with iCRT14 and subjected the cells to IR (4 Gy). Then, cells were subjected to IF staining for γH2AX. At the early time point (0.5 h after IR), no difference in the DNA damage foci formation was observed, suggesting that Wnt signalling activity is not associated with the early stages of DNA damage foci formation. However, at the later time point (24 h after IR), iCRT14-treated HCT116 cells still exhibited sustained γH2AX foci (Fig. 3a,b).

To complement this approach, we also activated Wnt/β-catenin signalling in human IECs (FHC and CCD841CoN) by treating the cells with Wnt3A (200 ng ml^−1^; 24 h) and subsequently with IR (4 Gy). After 24 h, we monitored γH2AX foci by IF staining. In contrast to the sustained γH2AX in the iCRT14-treated CRC cells, Wnt-activated IECs had fewer γH2AX foci than cells that were not treated with Wnt3A (Fig. 3c,d).

To further determine whether Wnt signalling-induced radioresistance is due to *LIG4* upregulation, we used SCR7, which inhibits LIG4 by interfering in the interaction between LIG4 and DNA^48^. We asked whether SCR7 treatment restores radiosensitization of IECs treated with Wnt3A. Indeed, SCR7 treatment increased γH2AX foci formation in CCD841CoN cells treated with Wnt3A and subsequent IR (Fig. 3e,f). These results suggest that Wnt signalling induces radioresistance via LIG4.

Next, we used plasmid recircularization assays to examine the effects of inhibiting Wnt signalling on NHEJ activity. Linearized plasmids encoding tdTomato-CreERT2 (TCE)-expressing plasmids were co-transfected with cyan fluorescent protein (CFP)-expressing plasmids (internal control) in SW620-7TGP GFP^High^ cells treated with dimethylsulfoxide (vehicle control) or iCRT14 (Fig. 3g). We found that iCRT14-treated SW620 GFP^High^ cells had lower tdTomato expression than the control cells (Fig. 3h,i). These results suggest that β-catenin enhances NHEJ in CRC cells.

We next investigated whether ectopic expression of LIG4 rescues Wnt signalling inhibition-induced defects in DSB repair. We established HCT116 cells that stably expressed LIG4 (wild type or R278H, a functionally inactive hypomorphic mutant)^49^. These HCT116 cells were treated with iCRT14 (50 μM for 24 h pretreatment) and IR (4 Gy) for γH2AX IF staining. While the control HCT116 cells pretreated with iCRT14 consistently showed the sustained γH2AX foci, iCRT14-treated HCT116 cells ectopically expressing LIG4 (HCT116-LIG4) exhibited significantly lower DNA damage foci formation (Fig. 3j,k). However, the R278H LIG4 mutant did not rescue Wnt signalling inhibition-induced radioresistance: these cells did not show any decrease in γH2AX foci formation 24 h after IR (Fig. 3j,k). Furthermore, ectopic expression of wild-type LIG4 but not R278H mutant recovered cell survival co-treated with iCRT14 and IR (Fig. 3l). These results suggest that *LIG4* mediates Wnt signalling-induced radioresistance.

### LIG4 expression in intestinal crypts

**Figure 4:**
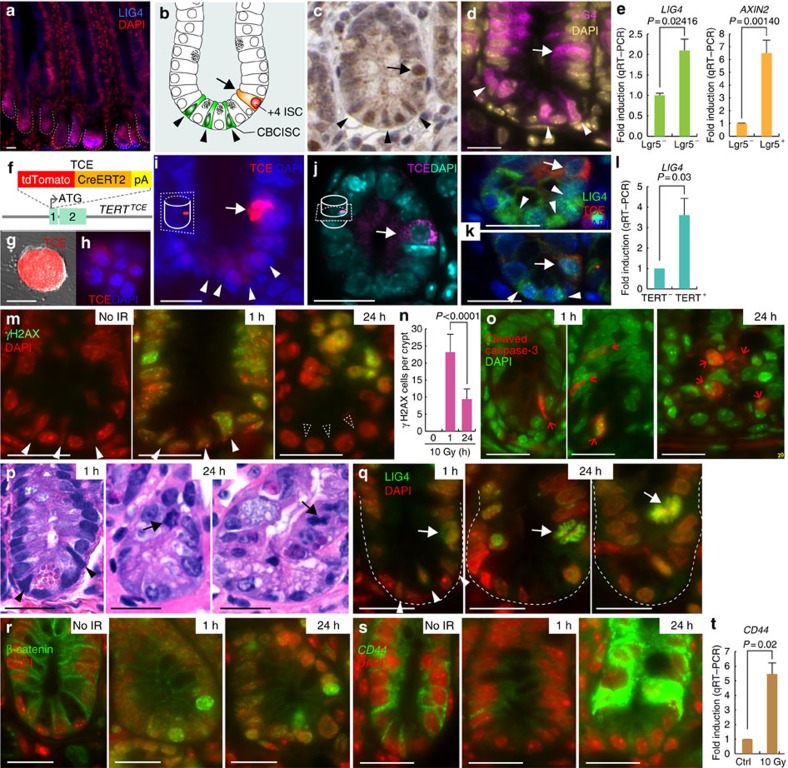
Expression of LIG4 in the intestine. (**a**) Expression of LIG4 in mouse small intestine samples; IF staining. Scale bars, 50μm. (**b**) Illustration of ISCs in the small intestine. ISCs located at position 4 (+4) are indicated by arrows, and CBC ISCs are indicated by arrowheads hereafter. (**c**) Expression of LIG4 in the human small intestine. 3,3′-Diaminobenzidine substrate staining. (**d**,**e**) Expression of LIG4 in the crypt of the mouse small intestine. IF staining (**d**) and qRT–PCR (**e**) of Lgr5^+^ and Lgr5^−^ cells isolated from the crypts of *Lgr5CreERT2* strain. *AXIN2* served as a positive control for Wnt/β-catenin signalling activity. Student's *t*-test; *N*=3; error bars=±s.e.m.; scale bars=20μm. (**f**) Generation of the *TERT*^*TCE*^ strain. TCE was inserted into the *TERT* allele-coding sequence in frame. (**g**,**h**) Expression of TCE in mouse ESCs. Low (**g**) and high (**h**) magnification. Scale bars=100μm. (**i**,**j**) TCE expression in the crypt of mouse small intestine samples (*TERT*^*TCE*^ mouse). Sagittal (**i**) and transverse (**j**) sections. Scale bars=20μm. (**k**,**l**) Expression of LIG4 in the TCE-expressing cells in the crypt of mouse small intestine samples (*TERT*^*TCE*^ mouse). IF staining (**k**) and qRT–PCR (**l**) of TERT^+^ cells isolated from *TERT*^*TCE*^. Student's *t*-test; *N*=3; error bars=±s.e.m.; scale bars=20μm. (**m**,**n**) WBI-induced DNA damage formation. Mice were treated with WBI (10 Gy; hereafter). Notably, CBC ISCs (arrowheads) disappeared 24 h after WBI. IF staining (**m**); quantification of phospho-γH2AX^+^ cells per crypts (the number of crypts counted ≥40) (**n**). Student's *t*-test; *N*=3; error bars=±s.e.m.; scale bars=20μm. (**o**) Apoptosis of CBC cells. Samples of mouse small intestine treated with WBI. Scale bars=20μm. (**p**) Mitosis of IECs after WBI. Mitotic cells (arrows); haematoxylin and eosin staining. Scale bars=20μm. (**q**) LIG4 upregulation in mitotic cells (arrows). CBC ISCs (arrowheads). Scale bars=20μm. (**r**) WBI-induced activation of β-catenin. Of note, WBI (10 Gy) rapidly induces β-catenin's localization change from the cell adhesion to the cytosol and the nucleus 1 and 24 h after WBI. Scale bars=20μm. (**s**,**t**) *CD44*, a β-catenin target gene, upregulation by WBI. One hour after WBI, *CD44* expression is diffused. Twenty-four hours after WBI, *CD44* expression is considerably upregulated in IECs at transit-amplifying zone but not in Paneth cells. IF staining (**s**); qRT–PCR of crypts (10 Gy, 24 h). Student's *t*-test; *N*=3; error bars=±s.e.m.; scale bars=20μm. For comparative analysis, images for each figure were captured under the same exposure time. DAPI, 4,6-diamidino-2-phenylindole.

Given that Wnt/β-catenin signaling upregulates *LIG4*, we further analysed the expression of LIG4 in the small intestine, where Wnt signalling is constitutively active for IEC replenishment. Although LIG4 is ubiquitously expressed in IECs, we found relatively high LIG4 expression in the intestinal crypts, where Wnt signalling is active (Fig. 4a). The intestinal crypts contain two distinct ISCs: quiescent ISCs (arrow), located at position 4 (+4), and highly proliferative crypt base columnar (CBC) ISCs (arrowheads; Fig. 4b). In human small intestine samples, LIG4 expression was detected in both quiescent and proliferative ISCs and in Paneth cells, IECs residing in the crypt bottom for secretion of anti-microbial compounds (Fig. 4c). IF staining of mouse small intestine samples showed that LIG4 expression in both types of ISCs was slightly higher than that in Paneth cells (Fig. 4d). qRT–PCR results further showed that Lgr5, a marker for CBC ISCs, -expressing cells isolated from the crypts of *Lgr5CreERT2* strain displayed the higher expression of *LIG4* than that in Lgr5^−^ cells (Fig. 4e). ISCs at position 4 are quiescent and contribute to intestinal regeneration, while CBC ISCs are constitutively proliferative during intestinal homeostasis^50^,^51^. However, a recent study also showed that CBC ISCs (Lgr5^+^) are indispensable for intestinal regeneration^52^. In addition, it was suggested that Bmi1, a marker for quiescent ISCs, is expressed broadly in the intestinal crypts rather than specifically in ISCs at position 4 (ref. 53).

Owing to this complicated issue, we used a functional stem cell marker to visualize ISCs. To make a stem cell reporter strain, we chose the telomerase reverse transcriptase (*TERT*) allele that encodes a catalytic subunit of telomerase, which is specifically expressed in self-renewing cells. By conventional gene targeting, we inserted the TCE cassette into the *TERT* coding sequence in frame (*TERT*^*TCE*^ knock-in strain), as previously performed^54^,^55^ (Fig. 4f and Supplementary Figs 5 and 6). We found that TCE was specifically expressed in mouse ESCs (Fig. 4g,h), validating TCE expression in self-renewing cells. Of note, TCE was detected only in the cytosol in the absence of tamoxifen, an activator of the CreERT2 fusion protein (Supplementary Fig. 6). In mouse small intestine, TCE was highly expressed in +4 ISCs and was mildly expressed in CBC ISCs (Fig. 4i,j). Next, we analysed the expression of LIG4 in *TERT*^*TCE*^ mouse small intestine samples by co-IF staining. We found that LIG4 expression is co-localized with TCE expression (Fig. 4k). Also, qRT–PCR showed that TERT^+^ cells isolated from *TERT*^*TCE*^ exhibited the higher expression of *LIG4* compared with that in TERT^−^ cells (Fig. 4l), indicating the enrichment of LIG4 in +4 TERT^+^ ISCs.

Next, we sought to determine how Wnt-induced LIG4 expression is involved in intestinal regeneration. We treated mice with whole-body IR (WBI, 10 Gy). After 1 or 24 h, the intestines were collected for immunohistochemistry. We observed that WBI induced acute γH2AX foci at 1 h after WBI. After 24 h, γH2AX foci disappeared in the crypt, whereas IECs at the crypt–villus boundary retained γH2AX foci (Fig. 4m,n). Moreover, we observed that CBC ISCs but not Paneth cells disappeared 24 h after WBI (Fig. 4m; dotted arrowheads). The loss of CBC ISCs was resulted from apoptosis, indicated by activation of caspase 3 in CBC ISCs (Fig. 4o). Moreover, 24 h after WBI, we found that cells in position 3–4 of the crypts had started to divide (Fig. 4p; arrows). Interestingly, we found that WBI-induced upregulation of LIG4 mainly occurred in these mitotic cells (Fig. 4q; arrows).

Given that β-catenin upregulates *LIG4* expression, we also asked whether WBI triggers β-catenin activation. In normal IECs, β-catenin is mainly associated with cell adhesion through E-cadherin. Surprisingly, after WBI, β-catenin was rapidly localized in the cytosol in IECs and in the nucleus of IECs at position 2–4 (Fig. 4r). We next assessed the expression of CD44, a β-catenin target gene in crypts specifically expressed on the cell surface of intestinal crypts. One hour after WBI, CD44 displayed the diffused pattern, and 24 h after WBI, *CD44* was markedly upregulated in IECs in position 3–4 but not in Paneth cells (Fig. 4s), recapitulated by qRT–PCR of *CD44* (Fig. 4t). With the immediate loss of cell adhesion-localized β-catenin and CD44 by IR (Fig. 4r–t), we also observed the localization change of p120-catenin, another catenin protein directly associated with the cell adhesion (E-cadherin), into the cytosol in an IR-treated mouse small intestine (Supplementary Fig. 7A), which led us to test whether IR induces mislocalization of the E-cadherin, an epithelial cell adhesion molecule. Indeed, we found the deceased E-cadherin expression in the IR-treated mouse small intestine (Supplementary Fig. 7B,C), which implies that the loss of E-cadherin by IR may release β-catenin into the cytosol for subsequent activation of LIG4. These results suggest that *LIG4* upregulated by Wnt/β-catenin signalling is relatively localized in the intestinal crypts, specifically in telomerase-expressing ISCs, which may serve as reservoirs for intestinal progenitor cells during intestinal regeneration on DNA damage.

### Expression of LIG4 in CRC

**Figure 5:**
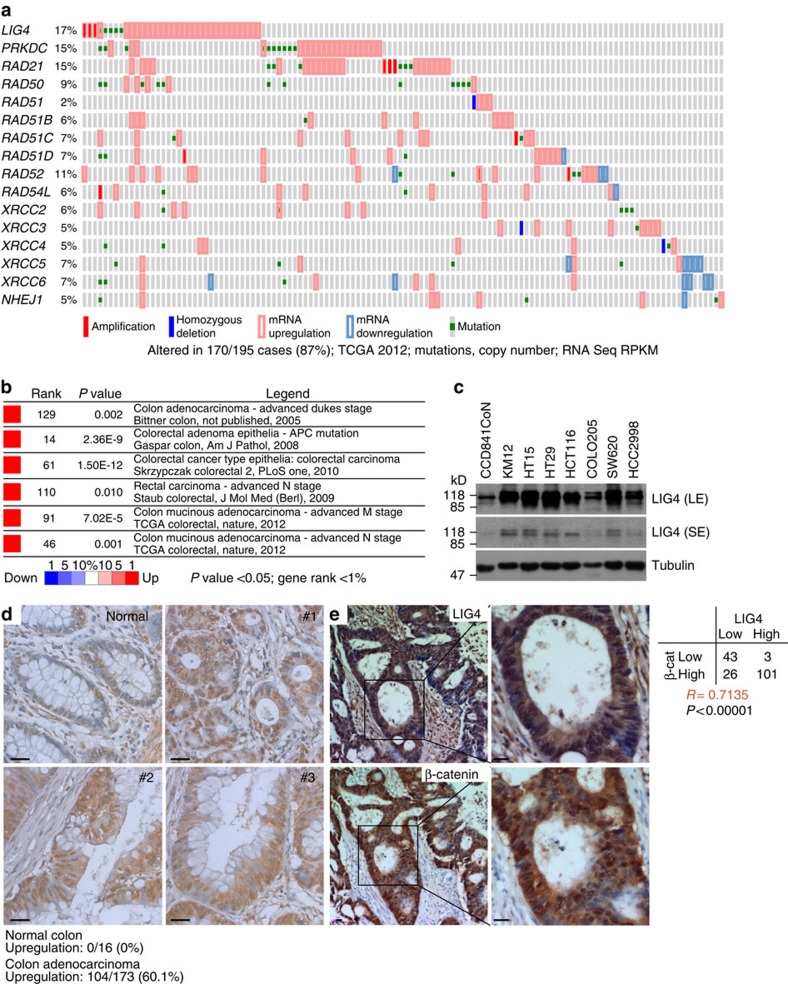
Upregulation of LIG4 in CRC cells. (**a**) cBioPortal analysis of the expression of NHEJ repair genes in CRC cells. Compared with the other NHEJ genes, *LIG4* is highly upregulated in CRC (17%). The cancer genome atlas (TCGA) 2012; 195 cases. (**b**) Oncomine analysis of *LIG4* expression in CRC cells. *P*<0.05; fold change >2. (**c**) Expression of LIG4 in CRC cell lines. Immunoblot analysis of LIG4 in CCD841CoN IECs and CRC cell lines. (**d**) Upregulation of LIG4 in CRC cells. Immunostaining of human CRC tissue microarray samples for LIG4; 3,3′-diaminobenzidine substrate staining. Human colorectal adenocarcinoma samples (#1–3). Scale bars=20μm. (**e**) Correlation between LIG4 and β-catenin upregulation. Immunostaining of human CRC tissue microarray for LIG4 and β-catenin (β-cat). Pearson correlation coefficient was calculated. *R*=0.7135; *P*<0.00001; scale bars=20μm.

Given that Wnt/β-catenin-induced *LIG4* mediates radioresistance in CRC cells, we next examined the expression of LIG4 in CRC. Using publicly available databases, we assessed the expression of DSB repair-associated genes in CRC. Interestingly, our cBioPortal database (cbioportal.org) analysis results indicated that *LIG4* is highly upregulated in CRC cells (17% with gene amplification and mRNA upregulation). Also, *PRKDC* and *RAD21* showed upregulated expression in CRC (15% for both). However, other DSB repair genes were not upregulated or genetically altered in CRC (Fig. 5a). Similarly, Oncomine database (oncomine.org) analysis results showed that *LIG4* expression is highly upregulated in CRC cells (1% top gene ranked), compared with normal colon tissues (Fig. 5b).

To confirm the upregulation of LIG4 protein in CRC, we assessed LIG4 expression in human IEC and CRC cell lines. Consistent with *in silico* results, immunoblot assays showed significant upregulation of LIG4 in all CRC cell lines, compared with IECs (CCD841CoN) (Fig. 5c). We further analysed the expression of LIG4 using a human CRC tissue microarray. As observed in mouse small intestine samples (Fig. 3), we found that human colonic crypts showed endogenous expression of LIG4 in the nucleus (Fig. 5d). However, human colorectal adenocarcinoma samples (*N*=173) displayed upregulation (60.1%) of LIG4 expression, compared with normal colorectal samples (Fig. 5d). Owing to *LIG4* upregulation by β-catenin, we examined the correlation between β-catenin and LIG4 expression in CRC TMA samples. We found that LIG4 upregulation is correlated with β-catenin upregulation in CRC (*R*=0.7135; Fig. 5e). These results suggest that LIG4 expression is highly upregulated in CRC cells, compared with normal IECs.

### Radiosensitization of CRC cells by inhibiting LIG4

**Figure 6:**
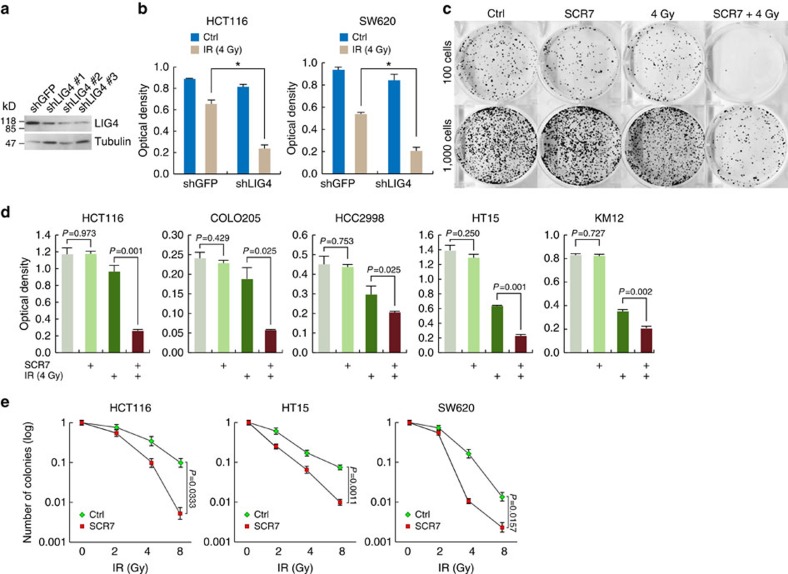
Radiosensitization of CRC cells by blockade of LIG4. (**a**) Depletion of endogenous LIG4 using shRNAs. HCT116 cells were stably transduced by lentiviruses encoding shRNAs against LIG4. Immunoblot assays. (**b**) Inhibition of cell survival by LIG4 depletion. HCT116 and SW620 (shGFP (control) and shLIG4) were treated with IR (4 Gy). Fourteen days after IR, cell survival was quantified by crystal violet staining. Student's *t*-test; *N*=3; *P<0.05; error bars=±s.e.m. (**c**,**d**) Decreased colony survival of CRC cells treated with SCR7. CRC cells were treated with SCR7 (10μM), were subjected to IR (4 Gy), and were grown for 14 days. Crystal violet staining of HCT116 cells (**c**) and quantification of colony survival of CRC cell lines based on optical density (**d**). Student's *t*-test; *N*=3; error bars=±s.e.m. (**e**) Radiosensitization by SCR7. CRC cells (HCT116, HT15 and SW620) pretreated with SCR7 (10μM, 24h) were treated with IR (0, 2, 4, and 8 Gy) and analyzed by clonogenic cell survival assays. Student's *t*-test; *N*=3; error bars=±s.e.m.

Given that *LIG4* is upregulated by β-catenin and is highly upregulated in CRC cells, we asked whether blocking LIG4 induces radiosensitization in CRC cells. We depleted the endogenous LIG4 using lentiviruses encoding shRNAs (Fig. 6a), then we checked cell survival on IR. LIG4-depleted HCT116 and SW620 cells display the decrease in cell survival on IR (Fig. 6b,c). Similarly, pretreatment of SCR7, an inhibitor of LIG4, followed by IR (4 Gy) induced the lower survival of CRC cell lines, compared with cells treated with IR only (Fig. 6d). Of note, SCR7 treatment *per se* did not affect cell survival (Fig. 6d, lane 2). Although SCR7 was previously shown to block tumour progression by inhibiting NHEJ^48^, the impact of SCR7 on CRC cells has not been tested. Thus, we asked whether SCR7 induces radiosensitization of CRC cells. Clonogenic cell survival assays showed that SCR7-treated HCT116, HT15 and SW620 CRC cells exhibited the radiosensitization (Fig. 6e). These results strongly suggest that blockade of LIG4 induces radiosensitization of CRC cells.

## Discussion

**Figure 7:**
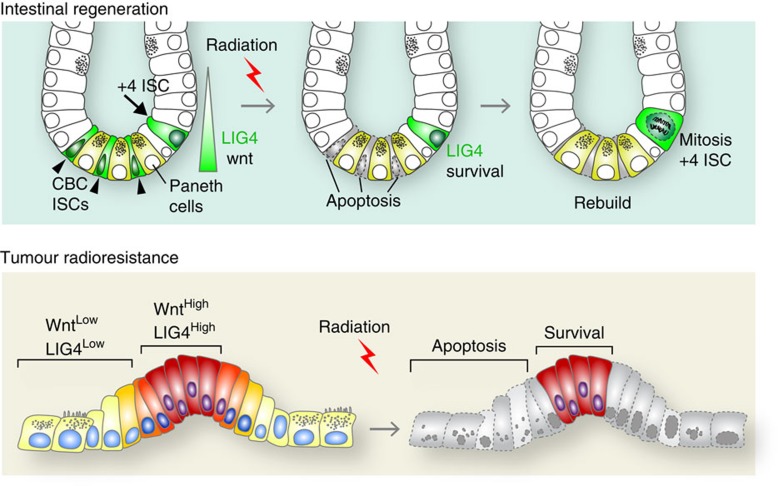
Model of Wnt/radiosensitization by SCR7. In the normal intestine, LIG4 expression is upregulated in intestinal crypts, where Wnt signalling is active. After genotoxic stress (IR), highly proliferative CBC ISCs undergo apoptosis. By an unknown mechanism, IR activates β-catenin, which leads to the upregulation of *LIG4* during intestinal regeneration.

Wnt signalling plays key roles in radioresistance during tissue regeneration and tumorigenesis. However, the molecular mechanism of Wnt signalling-mediated radioresistance remains unknown. We determined that LIG4, a key enzyme for NHEJ, mediates Wnt signalling-induced radioresistance in CRC cells and ISCs (Fig. 7).

We found that among the genes that participate in DSB repair, *LIG4* is upregulated by β-catenin in CRC cells and IECs. Of note, although blocking Wnt signalling significantly downregulates *LIG4*, inhibiting β-catenin does not completely suppress *LIG4* expression in CRC cells (Fig. 2c). This suggests that Wnt/β-catenin signalling enhances *LIG4* expression but is not required for basal-level expression of *LIG4*. This result is also supported by our finding that LIG4 was ubiquitously expressed in IECs but was highly enriched in the intestinal crypts where Wnt signalling is constitutively active (Fig. 4). Consistent with these results, we also observed that LIG4 was significantly upregulated in human CRC cells (Fig. 5). Furthermore, we found that blocking LIG4 sensitized CRC cells to IR (Fig. 6). These results strongly suggest that LIG4 mediates Wnt signalling-induced radioresistance in CRC cells.

One study suggested that DSB repair is a determinant of cellular radiosensitivity in radiation therapy. For DSB repair, mammalian cells dominantly use NHEJ more than HR, which led us to focus on NHEJ in Wnt signalling-controlled radioresistance. While HR occurs in the S and G2 phases of the cell cycle, NHEJ takes place throughout the cell cycle. Owing to the role of Wnt signalling in promoting cell proliferation via transactivation of *CCND1* and *MYC*, it is plausible that Wnt signalling-induced cell hyperproliferation may increase HR activity rather than NHEJ. However, CRC cells exhibiting different Wnt signalling activity (GFP^High^ versus GFP^Low^) did not show significant changes in cell proliferation (Supplementary Fig. 2). Thus, it is unlikely that the cell proliferation is involved in Wnt signalling-controlled radioresistance.

Intriguingly, we also observed that the expression of *PRKDC* and *RAD21* is highly upregulated in CRC cells (Fig. 5a). Although these DNA repair genes are not modulated by Wnt/β-catenin signalling, it is plausible that upregulation of these genes may also participate in radioresistance of CRC cells, which is supported by that the upregulation of these genes are mutually exclusive to that of *LIG4* (Fig. 5a). Therefore, our working model does not completely exclude the involvement of additional DNA repair genes in radioresistance of CRC cells.

In addition, it has been well established that p53 signalling functions as a gatekeeper for the initial DNA damage response, and via the p53 pathway. *LIG4* knockout induces neurogenesis defects and embryonic lethality^56^. However, our results showed that inhibiting Wnt/β-catenin signalling induces radiosensitization, independently of the p53 genetic status. For example, blocking Wnt signalling induces radiosensitization in both the p53 mutant cell lines (SW620, COLO205 and HCC2998) and the p53 wild-type cell line (HCT116). Thus, our study excludes the involvement of p53 or cell-cycle-mediated DNA repair in Wnt signalling-mediated radioresistance in CRC cells.

Previous studies showed that Wnt signalling contributes to radioresistance in normal IECs^9^,^10^. Our finding that Wnt/β-catenin-mediated *LIG4* transactivation induces radioresistance reveals the molecular mechanism of radioresistance in IECs. Interestingly, we found that LIG4 expression is relatively higher in CBC ISCs and +4 ISCs to terminally differentiated IECs (Fig. 4). Several studies suggested that +4 ISCs function as a reservoir to repopulate CBC ISCs and progenitor cells during regeneration^13^,^50^,^51^. Conversely, genetic cell targeting approaches showed that Lgr5^+^ ISCs are indispensable for IR-induced intestinal regeneration^52^. However, it should be carefully noted that unlike nonproliferating cells such as quiescent ISCs or terminally differentiated IECs, mitotic cells are hypersensitive to genotoxic stress, as also observed in our results (Fig. 4o). While +4 ISCs are quiescent until tissue regeneration is initiated^50^, CBC ISCs are constitutively proliferative, which may account for why CBC ISCs are hypersensitive to IR. Further studies are needed to determine how +4 ISCs maintain genomic stability and become mitotic during intestinal regeneration.

Owing to the complexity of defining ISCs^53^, we used our genetically engineered mouse model to specifically visualize self-renewing cells (*TERT*^*TCE*^). Importantly, we observed that LIG4 is relatively highly expressed in telomerase-expressing cells in the intestine, which implies that differentially expressed LIG4 may account for how radioresistant +4 ISCs maintain genomic integrity.

However, it is also noteworthy that both CBC and +4 ISCs exhibit high Wnt signalling activity. Thus, additional factor(s) may enhance or specify β-catenin-induced transactivation of *LIG4*. For example, we previously found that proliferating cell nuclear antigen (PCNA)-associated factor (PAF), a positive regulator of the β-catenin transcriptional complex, is specifically expressed in +4 ISCs and hyperactivates Wnt/β-catenin target gene activation^57^. Surprisingly, we found that LIG4 expression was significantly upregulated in mitotic cells after IR treatment, with overall stabilization and nuclear translocation of β-catenin and marked upregulation of *CD44* in IECs (Fig. 4q–t). Future studies are needed to determine how IR activates Wnt/β-catenin signalling.

Several studies have used a Wnt reporter system and shown that Wnt signalling activity identifies CRC stem-like cells^43^,^44^,^45^,^46^,^47^. Despite no difference in lentiviral DNA integration between clonally selected GFP^High^ (high Wnt activity) and GFP^Low^ (low Wnt activity) cells (Supplementary Fig. 8A), we found that GFP^High^ cells repopulate into GFP^High^ and GFP^Low^ cells, while GFP^Low^ cells divide into only GFP^Low^ cells, in a separate culture condition (Supplementary Fig. 8B). This stem cell-like property of GFP^High^ cells may be due to the (epi)genetic gene regulation or cell plasticity. Previously, we found that PAF functions as a cofactor of Wnt/β-catenin signalling in CRC^57^. Owing to specific expression of PAF in self-renewing cells, the heterogenous expression of PAF may contribute to the acquisition of stem cell-like property.

Although radiation therapy is a common cancer treatment, radioresistance in cancer cells is still a major limitation to overcome. To tackle this problem, we must understand the molecular mechanism of radioresistance, which will be beneficial for the development of a radiation sensitizer for cancer treatment. Furthermore, given that Wnt signalling-induced radioresistance is pivotal for tissue regeneration, understanding this mechanism would also provide valuable knowledge that could apply to the manipulation of tissue regeneration. Taken together, our results show the unexpected signalling convergence between Wnt signalling and DNA repair pathway, which holds great potential for translation into cancer treatment and regenerative medicine.

## Methods

### Mammalian cell culture and materials

CRC cell lines were purchased from the American Type Culture Collection and maintained in Dulbecco's modified Eagle medium (DMEM) containing 10% fetal bovine serum. CCD841CoN and FHC cells were cultured in DMEM-F12 containing 10% fetal bovine serum. Mycoplasma contamination was examined using MycoAlert mycoplasma detection kit (Lonza). iCRT14 and SRC7 were purchased from Santa Cruz Biotechnology and Xcess Biosciences, respectively. For depletion of endogenous LIG4, shLIG4 lentiviral plasmids (GIPZ; Open Biosystems) encoding shRNA were stably transduced. Three different LIG4 shRNAs were used (shLIG4 #1: 5′-GGATGATCATAAAGGATTT-3′; shLIG4 #2: 5′-CTATAATCCTAATACACAA-3′; and shLIG4 #3: 5′-TGGTGTTAGTCAGCAAACT-3′).

### Fluorescence-activated cell sorting

CRC cell lines were subjected to stable transduction with lentivirus encoding β-catenin reporter (7TGP; Addgene #24305). The cells were then sorted on the basis of GFP expression using FACSCalibur (Becton Dickinson FACSCalibur). Lgr5^+^ and TERT^+^ cells were isolated from the crypts of each mouse strain, as previously performed^57^. In brief, intestines from *Lgr5CreERT2* or *TTCE* mouse strains were washed in ice-cold phosphate-buffered saline (PBS) and subjected to removal of villi by scraping. To isolate Lgr5^+^ or TERT^+^ cells, the samples were incubated with 5 mM EDTA/PBS for 1 h at 4 °C on orbital shaker and followed by 10 min incubation in Accutase (Thermo/21-201-0100V) at 37 °C. Single-cell suspensions were isolated through 70μm (Biologix 15-1070) and 40 μm nylon mesh (BD Falcon #352340). Cells were stained with Sytox Blue(Invitrogen) for checking viability. In all, 200–500 viable EGFP (or tdTomato)-positive and -negative cells were sorted using FACS machine (MoFlo Astrios).

### Cell proliferation assays

Fourteen days after seeding the cells, we fixed the cells with 10% formalin and stained them with crystal violet for 30 min. For cell survival analysis, cells stained with crystal violet were subjected to lysis with 1% SDS, and absorbance was measured at 590 nm using a 96-well microplate reader (BioTek microplate reader).

### Clonogenic cell survival assays

Clonogenic cell survival assays were performed based on the routinely performed protocol. Briefly, before IR exposure, cells under different conditions including iCRT14 or SCR7 treatment, or GFP^High^ versus GFP^Low^ were trypsinized and plated (1 × 10^6^ cells per plate) to the triplicated 60-mm dish. After 24 h, the number of plated cells was counted to determine plating efficiency. On the basis of plating efficiency, the cells were plated and counted after 24 h for the confirmation of plating efficiency values. Depending on plating efficiency, the cells were plated to 60-mm dish and grown for 10–14 days. Finally, cells were fixed and stained with crystal violet. Colonies containing more than 50 cells were identified and scored as surviving colonies.

### PCR arrays

Repair gene expression in CRC cells was analysed with a Human DNA Repair RT^2^ Profiler PCR Array (QIAGEN).

### Gene expression analysis

For RNA extraction, cells were processed using TRIzol reagent (Invitrogen). RNA was subjected to reverse transcription using SuperScript II (Invitrogen). Next, complementary DNA was used for gene expression analysis using qRT–PCR. Hypoxanthine phosphoribosyltransferase 1 was used as an internal control for normalization. Fold induction was quantified using the 2^-ΔΔCT^ method, as previously described^57^. For gene expression analysis of sorted Lgr5^+^ and TERT^+^ cells, cDNA were generated using REPLI-g WTA Single Cell Kit (Qiagen #150063). qRT–PCR was performed using intron-spanning primers. Primer sequences are listed in Supplementary Table 1.

### *In silico* promoter analysis

The CNSs were analysed using the VISTA genome browser (http://www-gsd.lbl.gov/vista/), as previously performed^54^,^58^,^59^. Briefly, human and mouse LIG4 promoter was analysed with default options (200-bp window, *x* axis; 70% conservancy, *y* axis). If peak values (*y* axis) are above 50%, such promoter region (*x* axis) was considered as the evolutionarily conserved regulated elements.

### ChIP assays

ChIP assays were performed using β-catenin and TCF3 antibodies (Signal Transduction Laboratories). HCT116 cells were crosslinked with 1% formaldehyde for 15 min at room temperature. Formaldehyde was quenched by adding glycine (final concentration, 0.125 M). After washing the cells with cold 1 × PBS solution, we collected the cells with lysis buffer (0.5% NP-40, 25 mM HEPES, 150 mM KCl, 1.5 mM MgCl2, 10% glycerol and KOH (pH 7.5)) containing proteinase inhibitors and further incubated the cells on ice for 15 min. Cell lysates were centrifuged (5,000 r.p.m. for 5 min), and supernatants were discarded. Cell lysates were subjected to sonication with ChIP-radioimmunoprecipitation assay lysis buffer (50 mM Tris, pH 8.0; 150 mM NaCl; 0.1% SDS, 0.5% deoxycholate, 1% NP-40 and 1 mM EDTA; 10 times, 30 s on/30 s off) and were centrifuged (13,200 r.p.m. for 30 min). Supernatant from lysates was immunoprecipitated with antibody overnight at 4 °C and was pulled down using protein A/G PLUS-Agarose (Santa Cruz Biotechnology) by centrifugation (3,400 r.p.m. for 2 min). Immunoprecipitates were further washed serially with ChIP-radioimmunoprecipitation assay, high salt (50 mM Tris, pH 8.0; 500 mM NaCl; 0.1% SDS, 0.5% deoxycholate, 1% NP-40 and 1 mM EDTA), LiCl wash buffer (50 mM Tris, pH 8.0; 1 mM EDTA, 250 mM LiCl; 1% NP-40 and 0.5% deoxycholate) and Tris-EDTA buffer. Finally, immunoprecipitate crosslinking was reversed by incubation at 65 °C overnight, and immunoprecipitates were treated with RNase A and proteinase K to extract DNA. ChIP amplicons (1–8) were detected via ChIP-PCR. Primer sequences for ChIP-PCR are available in Supplementary Data 4.

### Establishment of *TERT* ^*TCE*^ genetically engineered mouse model

As previously described^54^, a conventional gene targeting method was employed to generate the *TERT*^*TCE*^ mouse model. As described in Supplementary Fig. 6, a targeting vector containing 5′ and 3′ homology arms for the *TERT* allele, the TCE cassette, the PGK-Neo positive selection cassette flanked by flippase recognition target (FRT) and the diphtheria toxin A (DTA) negative selection cassette was constructed. Then, the linearized targeting vector was electroporated into G4 mouse ESCs. After G418 selection, correctly targeted clones were identified by genotyping the 5′ and 3′ homology arms. Subsequently, targeted mouse ESCs were injected into blastocysts to generate chimeric mice that were further bred with the C57BL/6J strain for germ line transmission. Next, the G418 selection cassette was removed by the FLPeR deleter strain. *Lgr5CreERT2* strain was purchased from The Jackson Laboratories (008875). Lgr5^+^ cells were isolated from the crypts using FACS. Both male and female mice older than 4 weeks were used for experiments. All mice were treated in compliance with IACUC guideline of MD Anderson Cancer Center.

### cBioPortal and Oncomine database analysis

*LIG4* expression in CRC cells was analysed in the cBioPortal (www.cbioportal.org) and Oncomine (www.oncomine.org) databases, as previously described^57^. cBioPortal analysis was performed with default options using TCGA (2012) data sets for gene alterations (mutations and copy number change) and gene expression (RNA-Seq reads per kilobase million (RPKM)). For Oncomine analysis, the following options were chosen for analysis: *P*<0.05; gene rank <1%; and fold change >2.

### Immunofluorescent staining

Cells were fixed with 4% paraformaldehyde and were immunostained according to standard protocols: the cells were blocked and incubated with primary and fluorescence-conjugated secondary antibodies: LIG4 (Sigma (HPA001334); 1:250 dilution), and phospho-γH2AX (Cell Signaling (20E3); 1:250 dilution). The cells were stained with 4,6-diamidino-2-phenylindole. Cells that expressed phospho-γH2AX were examined with a fluorescent microscope (Zeiss). DSB foci were quantified using AxioVision software. Representative images are shown. For comparison, images were captured under the same exposure time.

### Immunohistochemistry

Mouse intestinal tissue samples were collected and fixed in 10% formalin and were processed for paraffin embedding. Sectioned samples were immunostained according to standard protocols: samples were deparaffinized, blocked and incubated with primary, and fluorescence- or horseradish peroxidase (HRP)-conjugated secondary antibodies. For HRP-conjugated secondary antibody, 3,3'-Diaminobenzidine substrate was used, followed by haematoxylin nuclear counterstaining. Antibodies against the following were used for immunohistochemistry: LIG4 (Sigma (HPA001334); 1:250 dilution); phospho-γH2AX (Cell Signaling (20E3); 1:250 dilution); RFP (Thermo (RF5R); 1:250 dilution); cleaved caspase 3 (Cell Signaling (5A1E); 1:500 dilution); β-catenin (Cell Signaling (D10A8); 1:500 dilution); *CD44* (BD Pharmingen (G44-26); 1:250 dilution); and *CD133* (eBioscience (13A4); 1:200 dilution). Immunostained tissues were photographed using a dissection microscope (Zeiss; AxioVision). For comparison, images were captured with the same exposure time. CRC tissue microarray slides were purchased from Biomax (Co2086).

### Immunoblotting assays

Whole-cell lysates were prepared using NP-40 lysis buffer (0.5% NP-40, 1.5 mM MgCl_2_, 25 mM HEPES, 150 mM KCl, 10% glycerol, 1 mM phenylmethylsulfonyl fluoride, 12.7 mM benzamidine HCl, 0.2 mM aprotinin, 0.5 mM leupeptin and 0.1 mM pepstatin A) for 20 min at 4 °C followed by centrifugation (14,000 r.p.m. for 10 min). Supernatants were denatured in 5 × SDS sample buffer (200 mM Tris-HCl, pH 6.8, 40% glycerol, 8% SDS, 200 mM dithiothreitol and 0.08% bromophenol blue) at 95 °C for 5 min followed by SDS–polyacrylamide gel electrophoresis. For immunoblot blocking and antibody incubation, 0.1% non-fat dry milk in Tris-buffered saline and Tween-20 (25 mM Tris-HCl, pH 8.0, 125 mM NaCl and 0.5% Tween-20) was used. SuperSignal West Pico and Femto reagents (Pierce) were used to detect HRP-conjugated secondary antibodies. The following antibodies were used for immunoblotting: LIG4 (Sigma (HPA001334); 1:5,000 dilution) and tubulin (Santa Cruz (A-6); 1:10,000). The original immunoblot images can be found in Supplementary Fig. 9.

### Plasmid recircularization assays

tdTomato-CreERT2-pcDNA3.1 plasmids were linearized by EcoRI located between the promoter and the coding sequence. CFP-pCMV plasmids were used as an internal control. Cells pretreated with dimethylsulfoxide (vehicle control) or iCRT14 were co-transfected with linearized tdTomato-CreERT2-pcDNA3.1 and CFP-pCMV (10:1 ratio). After 48 h, cells were fixed with 4% paraformaldehyde and were analysed by FACS.

### Statistical analysis

The Student's *t*-test was used for comparisons of two samples. *P* values <0.05 were considered significant. Error bars indicate s.e.m. The number of biological and experimental replicas ≥3, otherwise mentioned in figure legends.

## Additional information

**How to cite this article:** Jun, S. *et al*. *LIG4* mediates Wnt signalling-induced radioresistance. *Nat. Commun.* 7:10994 doi: 10.1038/ncomms10994 (2016).

## Supplementary Material

Supplementary Information Supplementary Figures 1-9 and Supplementary Table 1

Supplementary Data 1 Genes upregulated by β-catenin. 465 genes were identified as upregulated genes by β-catenin. Fold change > 2; P < 0.05.

Supplementary Data 2 DNA repair PCR array results. HCT116 cells treated with DMSO (vehicle; control) or iCRT14 (50 μM; 24 hours).

Supplementary Data 3 Heat map analysis of DNA repair PCR array experiments. HCT116 cells treated with DMSO (vehicle; control) or iCRT14 (50 μM 24 hours).

## References

[b1] Clevers H., Loh K. M. & Nusse R. Stem cell signaling. An integral program for tissue renewal and regeneration: Wnt signaling and stem cell control. Science 346, 1248012 (2014). 25278615 10.1126/science.1248012

[b2] Clevers H. & Nusse R. Wnt/beta-catenin signaling and disease. Cell 149, 1192–1205 (2012). 22682243 10.1016/j.cell.2012.05.012

[b3] Polakis P. Wnt signaling in cancer. Cold Spring Harb. Perspect. Biol. 4, a008052 (2012). 22438566 10.1101/cshperspect.a008052 PMC3331705

[b4] Clements W. M., Lowy A. M. & Groden J. Adenomatous polyposis coli/beta-catenin interaction and downstream targets: altered gene expression in gastrointestinal tumors. Clin. Colorectal Cancer 3, 113–120 (2003). 12952568 10.3816/ccc.2003.n.018

[b5] Su L. K. . Multiple intestinal neoplasia caused by a mutation in the murine homolog of the APC gene. Science 256, 668–670 (1992). 1350108 10.1126/science.1350108

[b6] Conard R. A. Some effects of ionizing radiation on the physiology of the gastrointestinal tract: a review. Radiat. Res. 5, 167–188 (1956). 13350498

[b7] Mason K. A., Withers H. R., McBride W. H., Davis C. A. & Smathers J. B. Comparison of the gastrointestinal syndrome after total-body or total-abdominal irradiation. Radiat. Res. 117, 480–488 (1989). 2648450

[b8] Clevers H. The intestinal crypt, a prototype stem cell compartment. Cell 154, 274–284 (2013). 23870119 10.1016/j.cell.2013.07.004

[b9] Hai B. . Concurrent transient activation of Wnt/beta-catenin pathway prevents radiation damage to salivary glands. Int. J. Radiat. Oncol. Biol. Phys. 83, e109–e116 (2012). 22342093 10.1016/j.ijrobp.2011.11.062 PMC3340568

[b10] Zhao J. . R-Spondin1 protects mice from chemotherapy or radiation-induced oral mucositis through the canonical Wnt/beta-catenin pathway. Proc. Natl Acad. Sci. USA 106, 2331–2336 (2009). 19179402 10.1073/pnas.0805159106 PMC2650156

[b11] Chen M. S. . Wnt/beta-catenin mediates radiation resistance of Sca1+ progenitors in an immortalized mammary gland cell line. J. Cell Sci. 120, 468–477 (2007). 17227796 10.1242/jcs.03348

[b12] Bhanja P. . Protective role of R-spondin1, an intestinal stem cell growth factor, against radiation-induced gastrointestinal syndrome in mice. PLoS ONE 4, e8014 (2009). 19956666 10.1371/journal.pone.0008014 PMC2777375

[b13] Zhou W. J., Geng Z. H., Spence J. R. & Geng J. G. Induction of intestinal stem cells by R-spondin 1 and Slit2 augments chemoradioprotection. Nature 501, 107–111 (2013). 23903657 10.1038/nature12416 PMC3888063

[b14] Lento W. . Loss of beta-catenin triggers oxidative stress and impairs hematopoietic regeneration. Genes Dev. 28, 995–1004 (2014). 24788518 10.1101/gad.231944.113 PMC4018497

[b15] Chandra A. . PTH1-34 blocks radiation-induced osteoblast apoptosis by enhancing DNA repair through canonical Wnt pathway. J. Biol. Chem. 290, 157–167 (2015). 25336648 10.1074/jbc.M114.608158 PMC4281718

[b16] Chang H. W. . Wnt signaling controls radiosensitivity via cyclooxygenase-2-mediated Ku expression in head and neck cancer. Int. J. Cancer 122, 100–107 (2008). 17764107 10.1002/ijc.23069

[b17] Woodward W. A. . WNT/beta-catenin mediates radiation resistance of mouse mammary progenitor cells. Proc. Natl Acad. Sci. USA 104, 618–623 (2007). 17202265 10.1073/pnas.0606599104 PMC1766434

[b18] Zhang M., Atkinson R. L. & Rosen J. M. Selective targeting of radiation-resistant tumor-initiating cells. Proc. Natl Acad. Sci. USA 107, 3522–3527 (2010). 20133717 10.1073/pnas.0910179107 PMC2840501

[b19] Li Y. . Evidence that transgenes encoding components of the Wnt signaling pathway preferentially induce mammary cancers from progenitor cells. Proc. Natl Acad. Sci. USA 100, 15853–15858 (2003). 14668450 10.1073/pnas.2136825100 PMC307657

[b20] Li G. . MicroRNA-324-3p regulates nasopharyngeal carcinoma radioresistance by directly targeting WNT2B. Eur. J. Cancer 49, 2596–2607 (2013). 23583221 10.1016/j.ejca.2013.03.001

[b21] Li H. Z. . Identification of differentially expressed genes related to radioresistance of human esophageal cancer cells. Chin. J. Cancer 29, 882–888 (2010). 20868558 10.5732/cjc.010.10148

[b22] Che S. M., Zhang X. Z., Liu X. L., Chen X. & Hou L. The radiosensitization effect of NS398 on esophageal cancer stem cell-like radioresistant cells. Dis. Esophagus 24, 265–273 (2011). 21087344 10.1111/j.1442-2050.2010.01138.x

[b23] Kim Y. . Wnt activation is implicated in glioblastoma radioresistance. Lab. Invest. 92, 466–473 (2012). 22083670 10.1038/labinvest.2011.161

[b24] Kendziorra E. . Silencing of the Wnt transcription factor TCF4 sensitizes colorectal cancer cells to (chemo-) radiotherapy. Carcinogenesis 32, 1824–1831 (2011). 21983179 10.1093/carcin/bgr222 PMC3254167

[b25] Jackson S. P. & Bartek J. The DNA-damage response in human biology and disease. Nature 461, 1071–1078 (2009). 19847258 10.1038/nature08467 PMC2906700

[b26] Deriano L. & Roth D. B. Modernizing the nonhomologous end-joining repertoire: alternative and classical NHEJ share the stage. Annu. Rev. Genet. 47, 433–455 (2013). 24050180 10.1146/annurev-genet-110711-155540

[b27] Sonoda E., Takata M., Yamashita Y. M., Morrison C. & Takeda S. Homologous DNA recombination in vertebrate cells. Proc. Natl Acad. Sci. USA 98, 8388–8394 (2001). 11459980 10.1073/pnas.111006398 PMC37448

[b28] Williams G. J. . Structural insights into NHEJ: building up an integrated picture of the dynamic DSB repair super complex, one component and interaction at a time. DNA Repair (Amst) 17, 110–120 (2014). 24656613 10.1016/j.dnarep.2014.02.009 PMC4102006

[b29] Ochi T. . DNA repair. PAXX, a paralog of XRCC4 and XLF, interacts with Ku to promote DNA double-strand break repair. Science 347, 185–188 (2015). 25574025 10.1126/science.1261971 PMC4338599

[b30] O'Driscoll M. . DNA ligase IV mutations identified in patients exhibiting developmental delay and immunodeficiency. Mol. Cell 8, 1175–1185 (2001). 11779494 10.1016/s1097-2765(01)00408-7

[b31] Oh S., Wang Y., Zimbric J. & Hendrickson E. A. Human LIGIV is synthetically lethal with the loss of Rad54B-dependent recombination and is required for certain chromosome fusion events induced by telomere dysfunction. Nucleic Acids Res. 41, 1734–1749 (2013). 23275564 10.1093/nar/gks1326 PMC3561972

[b32] Nijnik A. . DNA repair is limiting for haematopoietic stem cells during ageing. Nature 447, 686–690 (2007). 17554302 10.1038/nature05875

[b33] Felgentreff K. . Differential role of nonhomologous end joining factors in the generation, DNA damage response, and myeloid differentiation of human induced pluripotent stem cells. Proc. Natl Acad. Sci. USA 111, 8889–8894 (2014). 24889605 10.1073/pnas.1323649111 PMC4066476

[b34] Tilgner K. . A human iPSC model of ligase IV deficiency reveals an important role for NHEJ-mediated-DSB repair in the survival and genomic stability of induced pluripotent stem cells and emerging haematopoietic progenitors. Cell Death iDffer. 20, 1089–1100 (2013). 10.1038/cdd.2013.44 PMC3705601 23722522

[b35] Munroe R. J. . Mouse mutants from chemically mutagenized embryonic stem cells. Nat. Genet. 24, 318–321 (2000). 10700192 10.1038/73563 PMC2868360

[b36] Thomas J. W., LaMantia C. & Magnuson T. X-ray-induced mutations in mouse embryonic stem cells. Proc. Natl Acad. Sci. USA 95, 1114–1119 (1998). 9448294 10.1073/pnas.95.3.1114 PMC18691

[b37] Tichy E. D. . The abundance of Rad51 protein in mouse embryonic stem cells is regulated at multiple levels. Stem Cell Res. 9, 124–134 (2012). 22705496 10.1016/j.scr.2012.05.004 PMC3412895

[b38] Harfouche G. & Martin M. T. Response of normal stem cells to ionizing radiation: a balance between homeostasis and genomic stability. Mutat. Res. 704, 167–174 (2010). 20117235 10.1016/j.mrrev.2010.01.007

[b39] Bao S. . Glioma stem cells promote radioresistance by preferential activation of the DNA damage response. Nature 444, 756–760 (2006). 17051156 10.1038/nature05236

[b40] Karimi-Busheri F., Rasouli-Nia A., Mackey J. R. & Weinfeld M. Senescence evasion by MCF-7 human breast tumor-initiating cells. Breast Cancer Res. 12, R31 (2010). 20525204 10.1186/bcr2583 PMC2917024

[b41] Zabludoff S. D. . AZD7762, a novel checkpoint kinase inhibitor, drives checkpoint abrogation and potentiates DNA-targeted therapies. Mol. Cancer Ther. 7, 2955–2966 (2008). 18790776 10.1158/1535-7163.MCT-08-0492

[b42] Fuerer C. & Nusse R. Lentiviral vectors to probe and manipulate the Wnt signaling pathway. PLoS ONE 5, e9370 (2010). 20186325 10.1371/journal.pone.0009370 PMC2826402

[b43] Vermeulen L. . Wnt activity defines colon cancer stem cells and is regulated by the microenvironment. Nat. Cell Biol. 12, 468–476 (2010). 20418870 10.1038/ncb2048

[b44] Evans J. . Registered report: Wnt activity defines colon cancer stem cells and is regulated by the microenvironment. Elife 4, e07301 (2015). 10.7554/eLife.07301 PMC4541490 26287525

[b45] de Sousa E. M. F. . Methylation of cancer-stem-cell-associated Wnt target genes predicts poor prognosis in colorectal cancer patients. Cell Stem Cell 9, 476–485 (2011). 22056143 10.1016/j.stem.2011.10.008

[b46] Sadanandam A. . A colorectal cancer classification system that associates cellular phenotype and responses to therapy. Nat. Med. 19, 619–625 (2013). 23584089 10.1038/nm.3175 PMC3774607

[b47] de Sousa E. M., Vermeulen L., Richel D. & Medema J. P. Targeting Wnt signaling in colon cancer stem cells. Clin. Cancer Res. 17, 647–653 (2011). 21159886 10.1158/1078-0432.CCR-10-1204

[b48] Srivastava M. . An inhibitor of nonhomologous end-joining abrogates double-strand break repair and impedes cancer progression. Cell 151, 1474–1487 (2012). 23260137 10.1016/j.cell.2012.11.054

[b49] Riballo E. . Identification of a defect in DNA ligase IV in a radiosensitive leukaemia patient. Curr. Biol. 9, 699–702 (1999). 10395545 10.1016/s0960-9822(99)80311-x

[b50] Yan K. S. . The intestinal stem cell markers Bmi1 and Lgr5 identify two functionally distinct populations. Proc. Natl Acad. Sci. USA 109, 466–471 (2012). 22190486 10.1073/pnas.1118857109 PMC3258636

[b51] Tian H. . A reserve stem cell population in small intestine renders Lgr5-positive cells dispensable. Nature 478, 255–259 (2011). 21927002 10.1038/nature10408 PMC4251967

[b52] Metcalfe C., Kljavin N. M., Ybarra R. & de Sauvage F. J. Lgr5+ stem cells are indispensable for radiation-induced intestinal regeneration. Cell Stem Cell 14, 149–159 (2014). 24332836 10.1016/j.stem.2013.11.008

[b53] Munoz J. . The Lgr5 intestinal stem cell signature: robust expression of proposed quiescent '+4' cell markers. EMBO J. 31, 3079–3091 (2012). 22692129 10.1038/emboj.2012.166 PMC3400017

[b54] Park J. I. . Telomerase modulates Wnt signalling by association with target gene chromatin. Nature 460, 66–72 (2009). 19571879 10.1038/nature08137 PMC4349391

[b55] Jung H. Y., Wang X., Jun S. & Park J. I. Dyrk2-associated EDD-DDB1-VprBP E3 ligase inhibits telomerase by TERT degradation. J. Biol. Chem. 288, 7252–7262 (2013). 23362280 10.1074/jbc.M112.416792 PMC3591633

[b56] Frank K. M. . DNA ligase IV deficiency in mice leads to defective neurogenesis and embryonic lethality via the p53 pathway. Mol. Cell 5, 993–1002 (2000). 10911993 10.1016/s1097-2765(00)80264-6

[b57] Jung H. Y. . PAF and EZH2 induce Wnt/beta-catenin signaling hyperactivation. Mol. Cell 52, 193–205 (2013). 24055345 10.1016/j.molcel.2013.08.028 PMC4040269

[b58] Jun S. . PAF-mediated MAPK signaling hyperactivation via LAMTOR3 induces pancreatic tumorigenesis. Cell Rep. 5, 314–322 (2013). 24209743 10.1016/j.celrep.2013.09.026 PMC4157353

[b59] Jung Y. S., Jun S., Lee S. H., Sharma A. & Park J. I. Wnt2 complements Wnt/beta-catenin signaling in colorectal cancer. Oncotarget 6, 37257–37268 (2015). 26484565 10.18632/oncotarget.6133 PMC4741928

