# Supplementary material for: LIG4 mediates Wnt signalling-induced radioresistance
Source: Nat Commun. 2016 Mar 24;7:10994. doi: 10.1038/ncomms10994 (PMC4820809; doi:10.1038/ncomms10994)
Supplement: Supplementary Information — Supplementary Figures 1-9 and Supplementary Table 1 [file ncomms10994-s1.pdf]

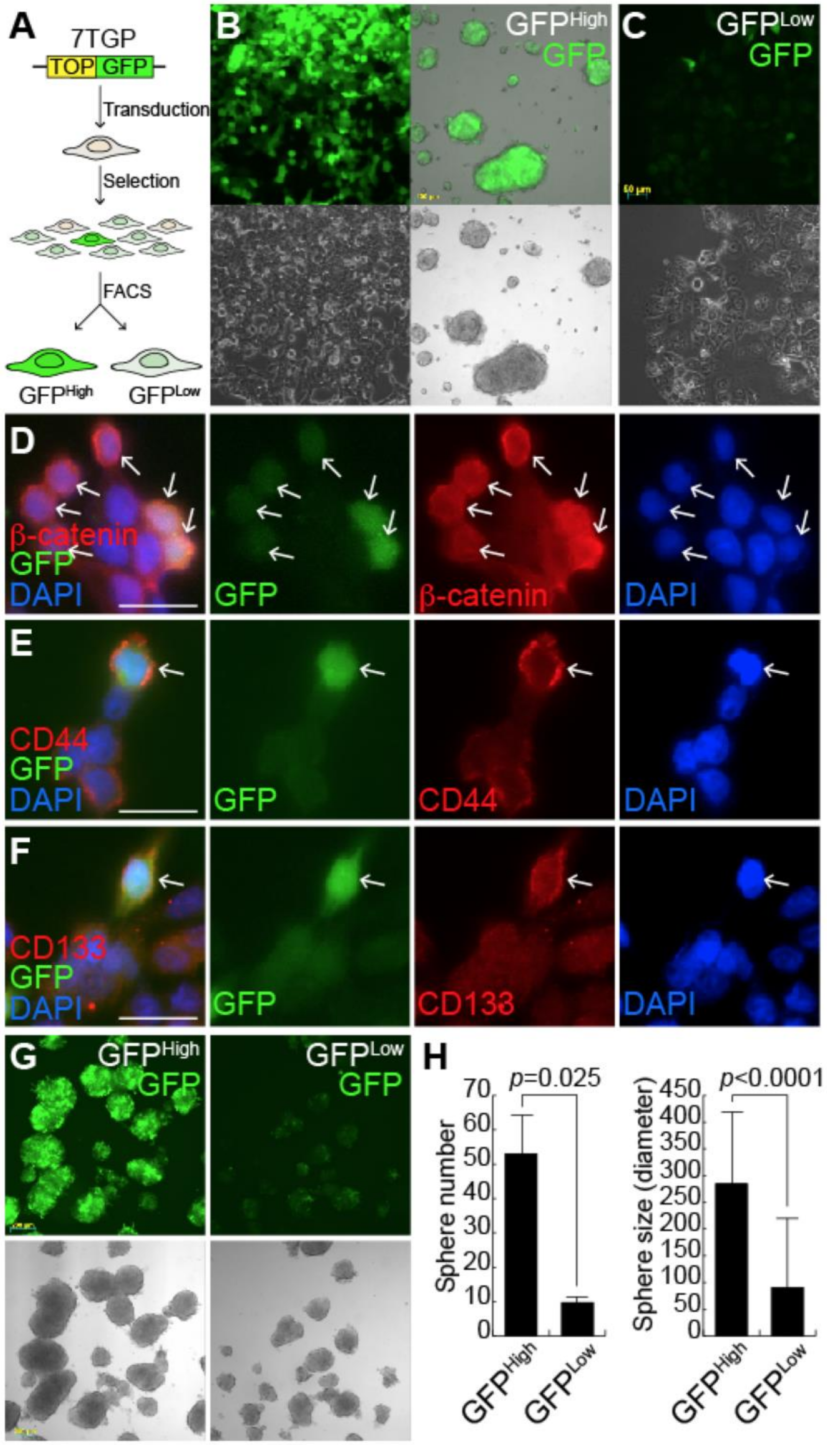

Supplementary Figure 1. Isolation of GFP^{High} cells. (A) Schematic diagram of cell isolation based on Wnt signaling activity. Colorectal cancer (CRC) cell lines were stably transduced with lentivirus encoding ionatenin reporter (7TGP). After selection, cells were processed for fluorescence-activated cell sorting (FACS), based on GFP expression. Parental HCT116 cells (left panel) served as negative controls for cell sorting. Scale bars = 100 µm.
(B and C) Isolation of GFP^{High} cells from CRC cells. After FACS, HCT116-7TGP cells were further cultured. GFP^{High} (B) and GFP^{Low} cells (C). Scale bars = 50 µm.
(D) Increased β-catenin expression in GFP^{High} cells. Immunofluorescent (IF) staining of SW620-7TGP cells. Scale bars = 20 µm. Arrows: cells highly expressing β-catenin.
(E and F) Upregulation of β-catenintarget genes in GFP^{High} cells. IF staining of SW620-7TGP cells for β-catenin (D), CD44 (E), and CD133 (F). Scale bars = 20 µm. Arrows: cells highly expressing CD44 or CD133.
(G and H) Increased sphere formation of GFP^{High} cells. GFP^{High} and GFP^{Low} HCT116-7TGP cells were grown in low attachment condition for 14 days. Images (G) and quantification of the number and size of spheres (H). Scale bars = 200 µm; Student's *t*-test; N = 3. Error bars = SEM.

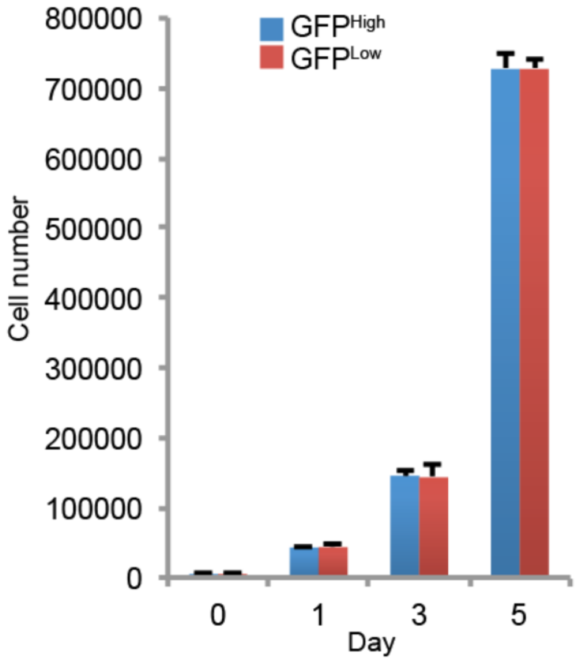

Supplementary Figure 2. No difference in cell proliferation between GFP^{High} and GFP^{Low} cells

HCT116-7TGP cells were sorted based on GFP expression using FACS. 5,000 cells were plated and counted at day 1, 3, and 5, using Biorad cell counter (TC10). Student's *t*-test; N = 3. Error bars = SEM.

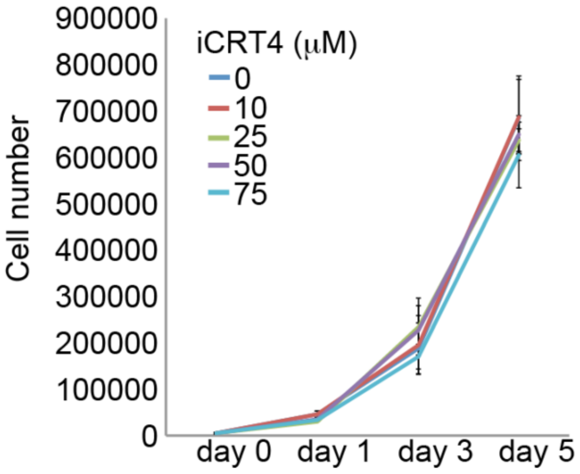

Supplementary Figure 3. No effects of iCRT14 on CRC cell proliferation.
HCT116 cells were treated with iCRT14 (0~75 μM) and analyzed for cell counting at day 1, 3, and 5. 5,000 cells were plated initially. Student's *t*-test; N = 3. Error bars = SEM.

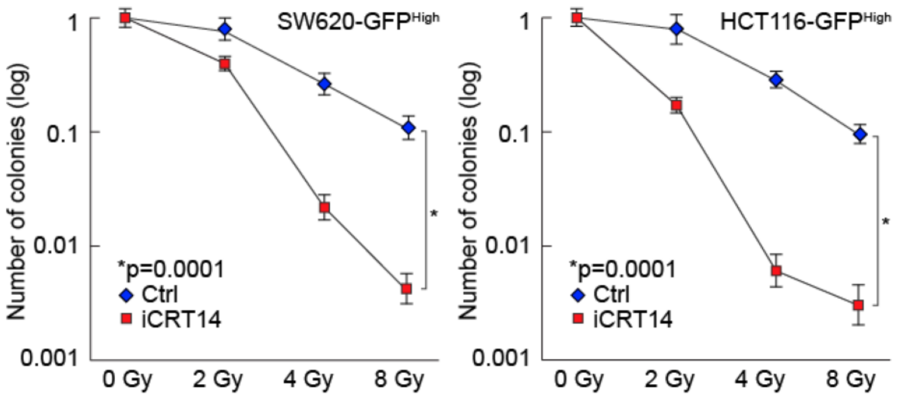

Supplementary Figure 4. Radiosensitization of GFP^{High} CRC cells by iCRT14. SW620-GFP^{High} and HCT116-GFP^{High} cells were analyzed for clonogenic assays under IR (0, 2, 4, and 8 Gy). Cells were continuously treated with iCRT14 (50 μM). Student's *t*-test; N = 3. Error bars = SEM.

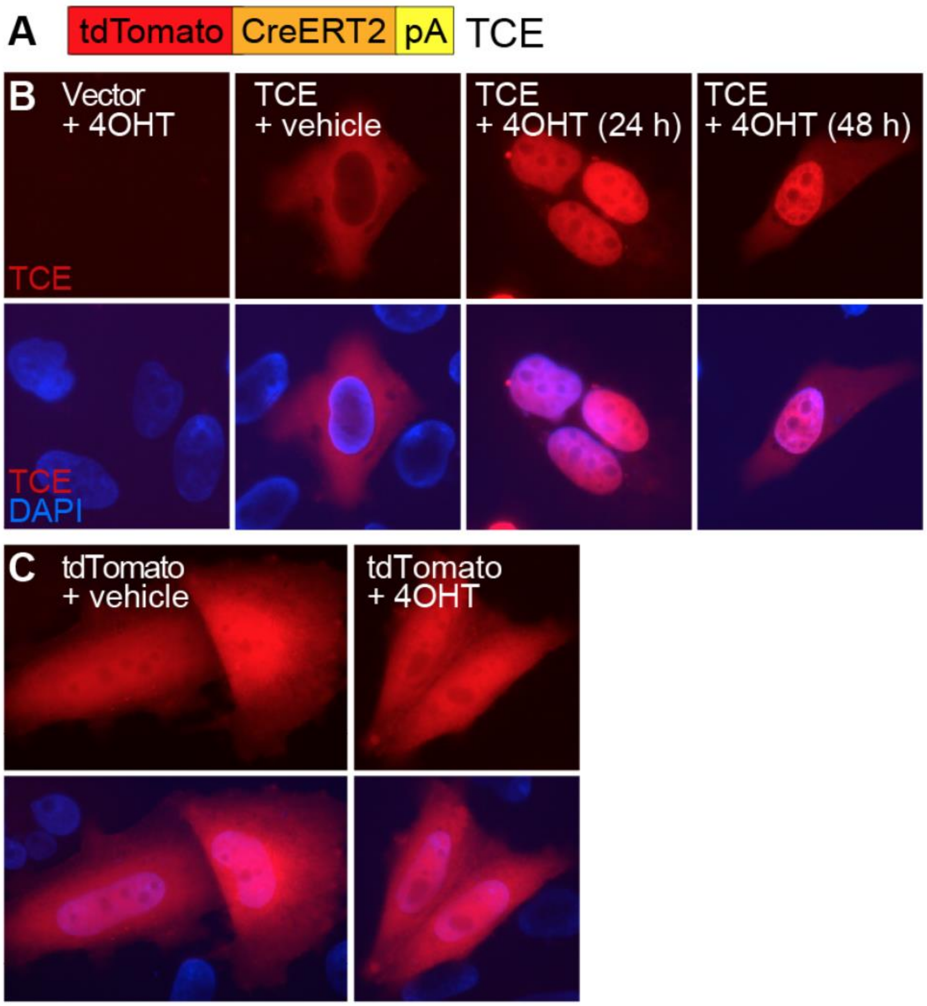

Supplementary Figure 5. Validation of TCE cassette
Prior to generating TERTTCE knock-in mESCs and mouse model, we characterized tdTomato-CreERT2 (TCE) cassette.
(A) Illustration of TCE fusion protein. pA: poly (A) signal.
(B and C) Nuclear translocation of TCE by 4-hydroxy-tamoxigen (4OHT) treatment. HeLa cells were transiently transfected with TCE-pcDNA3.1 plasmid. 24 hours after transfection, cells were treated with 100 μM 4OHT (in ethanol) and analyzed for IF staining of TCE (tdTomato red fluorescent signal). 24 hours after 4OHT treatment, TCE was completely localized in the nucleus. At 48 hours after 4OHT treatment, the cytosolic TCE was also observed (B). However, tdTomato (a negative control) displayed subcellular localization both in the nucleus and in the cytosol, regardless of 4OHT treatment (C).

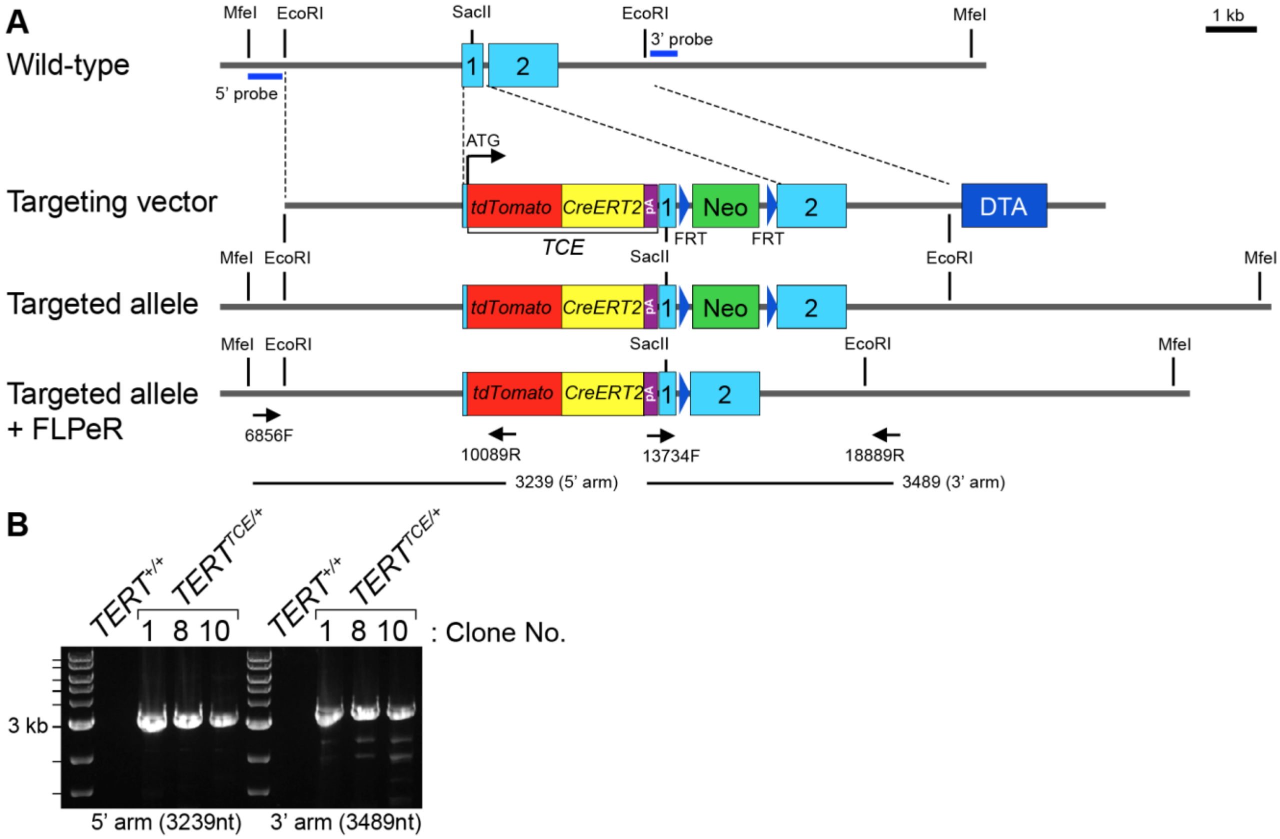

Supplementary Figure 6. Establishment of *TERT*^{TCE} genetically engineered mouse model

(A) Gene targeting (knock-in) strategy. We constructed targeting vector containing 5' homologous arm, tdTomato-CreERT2-pA, Neo cassette (for positive selection) flanked by FRT sites, 3' homologous arm, and diphtheria toxin A (DTA) (for negative selection) cassette. Of note, TCE cassette was inserted into *TERT* allele in-frame. Linearized targeting plasmids were electroporated into G4 mESCs to generate *TERT*$^{TCE\text{-}Neo}$ mESCs. mESCs were injected into the blastocysts to generate chimeric mice. Mice confirmed for germ line transmission were further bred with FLPeR deletor strain to remove Neo selection cassette (*TERT*^{TCE} strain).

(B) Genotyping of *TERT*^{TCE} strain. For genotyping, insertion of 5' and 3' homologous arms were confirmed by PCR of genomic DNA of *TERT*$^{TCE\text{-}Neo}$ mESCs. Clones #1, 8, and 10 were selected for blastocyst injection.

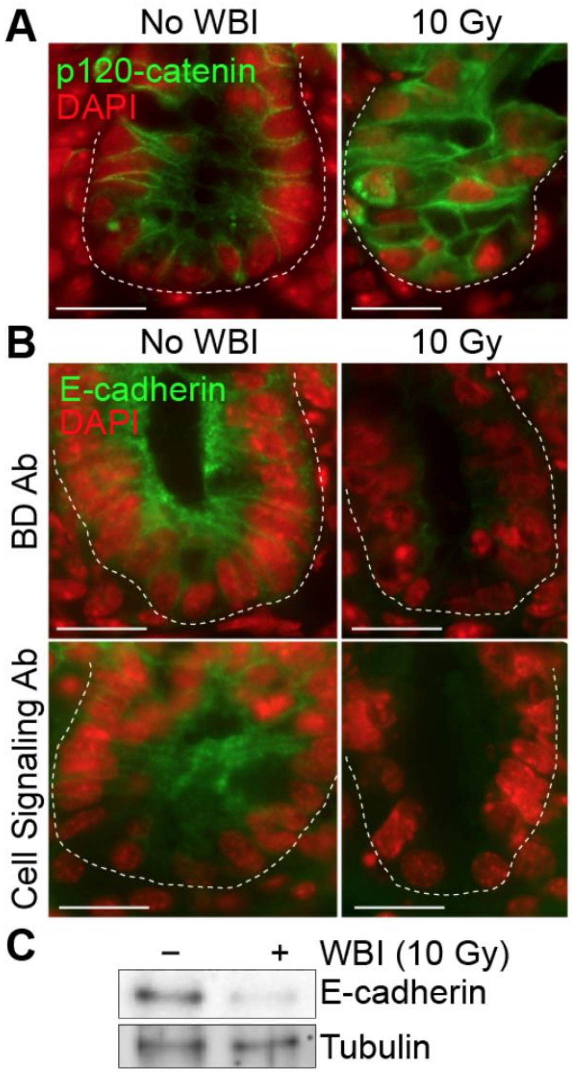

Supplementary Figure 7. Change in cell adhesion components by radiation
(A) Localization change of p120-catenin by radiation. Mice were treated with WBI (10 Gy). 24 hours later, mouse small intestine samples were collected for immunostaining for p120-catenin (Cell Signaling). Of note, in normal intestine, p120-catenin is specifically associated with E-cadherin. However, WBI-treated small intestine displays the cytosolic and the cell adhesion-associated p120-catenin.
(B and C) The decrease of E-cadherin by WBI. Untreated and WBI (10 Gy)-treated mouse small intestine samples were subjected to immunostaining of E-cadherin (B). Using two different antibodies for E-cadherin detection similarly exhibited the significant decrease of E-cadherin by WBI. Consistently, immunoblotting of intestinal epithelial cells isolated from mouse small intestine (control and WBI) also showed the decreased level of E-cadherin (C). Representative images were shown. All images were captured under the same exposure time for comparison. Scale bars = 20 μm.

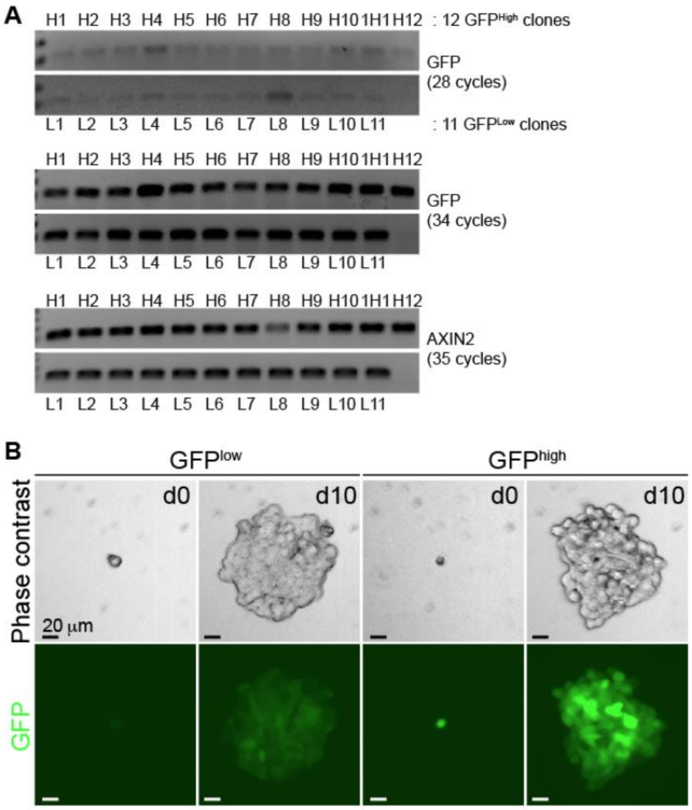

Supplementary Figure 8. Analysis of lentiviral reporter plasmid integration
(A) Quantification of lentiviral DNA integration. 12 GFP^{High} (H1~H12) and 11 GFP^{Low} (L1~L11) clones were clonally picked and cultured for genomic DNA (gDNA) extraction. 0.05 ug of gDNA was used for PCR. GFP PCR amplification is similar between 12 GFP^{High} and 11 GFP^{Low} clones at 28 and 34 PCR cycles. *AXIN2* promoter DNA was also amplified as internal controls.
(B) Expression of GFP in the sorted cells. SW620-7TGP cells were sorted by GFP expression (GFP^{high} and GFP^{low}). Then, clonally selected cells were grown from a single cell for 10 days, and analyzed by GFP expression (500 msec exposure). Scale bars = 20 μm

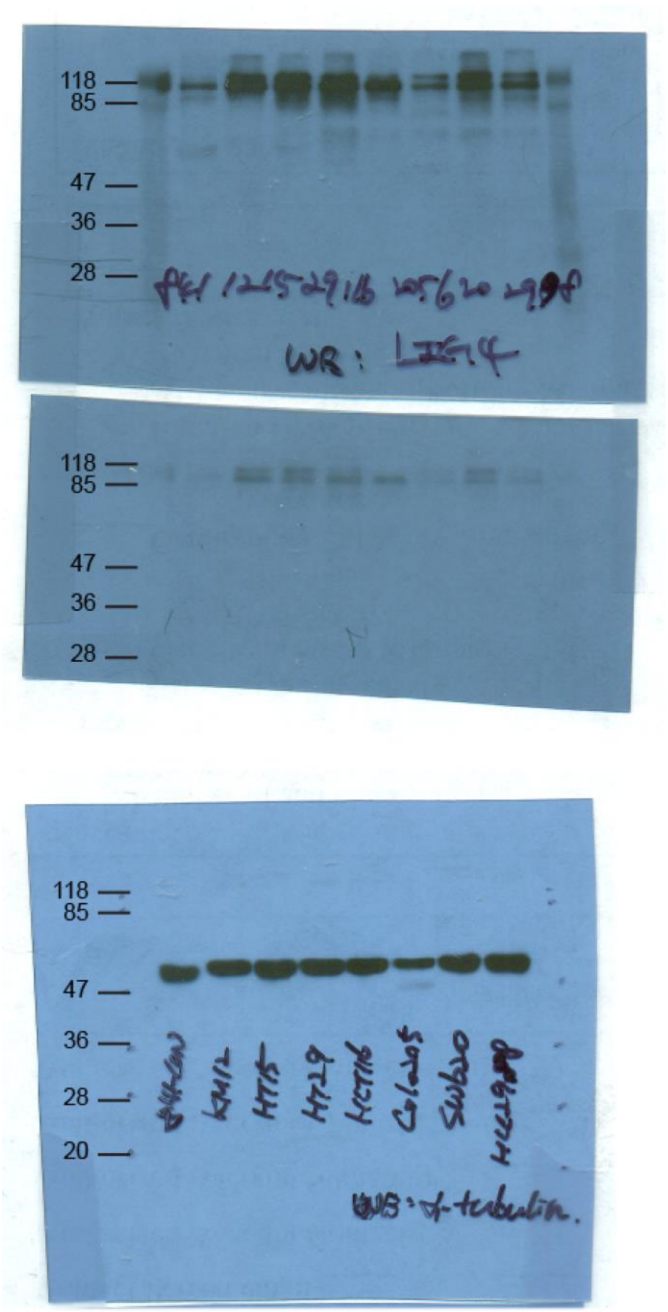

Supplementary Figure 9. Uncropped immunoblot images of Figure 5C.

Supplementary Table 1. Primer sequences for qRT-PCR and ChIP-PCR

**qRT-PCR**		from 5' to 3'
	PRKDC-F	AGGACTGGCGTGTGAAACTT
	PRKDC-R	ACAGCAGCATGTCATGGAAG
	RAD21-F	TGGCTGGCTATGAAAACAGA
	RAD21-R	CTGCTCGGAAGCTACACCTC
	RAD50-F	TCCACGATAGGTACTTCGCC
	RAD50-R	TGAGGACAACAGAACTTGTGAAC
	RAD51-F	GGTCTGGTGGTCTGTGTTGA
	RAD51-R	GGTGAAGGAAAGGCCATGTA
	RAD51C-F	GTGTGACTCAGATGTACCAGCA
	RAD51C-R	CTAGAGGTGAAACCCTCCGA
	RAD51B-F	GCTCCACTCAGATGGGTTGT
	RAD51B-R	GATGCACAACTTCAAGGCAA
	RAD51D-F	CCACATTTGCTGCCATACAG
	RAD51D-R	TACTGCCATCCTGTCCACTG
	RAD52-F	CTGGCACTGTCCAAAGCATA
	RAD52-R	TAGATCGAGCTCCCTGTGTG
	RAD54L-F	AGAGCCCAGAGGACCTTGAT
	RAD54L-R	TCCTTTTCGGAAACCTTTGA
	XRCC2-F	TCTACCTTCAAGTCGGGCAA
	XRCC2-R	TAGAGTCTGCGCAGTTGGTG
	XRCC3-F	CGTCTTCCGTGCAGATGTAG
	XRCC3-R	CATCACTGAGCTGGCCG
	XRCC4-F	TTTCAGCTGAGATGTGCTCC
	XRCC4-R	AGGAGACAGCGAATGCAAAG
	XRCC5-F	GAAGGCTCGGATGCAGTCTA
	XRCC5-R	CCTGCTGAAAACTTCCGTGT
	XRCC6-F	TGGTTCATTTGTTTCCCGAT
	XRCC6-R	AGACCAGGAAGCGAGCACT
	NHEJ1-F	TGCAGATTCATGACAAAGGG
	NHEJ1-R	ACTACCAGGAGAGTGGGGCT
**ChIP-promoter scanning**		from 5' to 3'
	LIG4ChIP-1F	AGTGATGGCCATGCTTCTCT
	LIG4ChIP-1R	GGGAGCCTGCAGTGATATTC
	LIG4ChIP-2F	AATGGGGTCCACAAGATCAG
	LIG4ChIP-2R	TTCCCTCATTTACCGTGAGC
	LIG4ChIP-3F	AACCCAGGAGTCGAGGTTG
	LIG4ChIP-3R	CATTTGCTGGTGGCAGAAT
	LIG4ChIP-4F	GCACAGCTGAAATGGAAACA
	LIG4ChIP-4R	GATGCTGTCTGAACCATCCA

LIG4ChIP-5F	CCCAGGGCAATTTGGTTAG
LIG4ChIP-5R	CACACTGCCTCCCACTGAC
LIG4ChIP-6F	CACCAGGACTCCCTCCAGT
LIG4ChIP-6R	TGCGACACCCTTAACAACC
LIG4ChIP-7F	TCCCTTGGGCTTTCCTTATT
LIG4ChIP-7R	AGAGGCTGAGGCGAGAGAAT
LIG4ChIP-8F	GGTCAAAGCTGGGTTCTTGA
LIG4ChIP-8R	TTCAAAGCCCAGTCTCCATT
